# Small molecule inhibitor targeting the Hsp70-Bim protein–protein interaction in estrogen receptor-positive breast cancer overcomes tamoxifen resistance

**DOI:** 10.1186/s13058-024-01790-0

**Published:** 2024-02-26

**Authors:** Ting Song, Hong Zhang, Qicheng Zhao, Zhiyuan Hu, Ziqian Wang, Yang Song, Zhichao Zhang

**Affiliations:** 1 Cancer Hospital of Dalian University of Technology, School of Chemistry, Dalian University of Technology, Dalian, Liaoning China; 2 Cancer Rehabilitation Center, Shanghai YangZhi Rehabilitation Hospital (Shanghai Sunshine Rehabilitation Center), School of Medicine, Tong Ji University, Shanghai, China; 3 School of Life Science and Technology, Dalian University of Technology, Dalian, Liaoning China; 4 Central Hospital of Dalian University of Technology, Dalian, Liaoning China

**Keywords:** Hsp70-Bim PPI, Hsp70-Bag3 PPI, Tamoxifen resistance, Estrogen receptor α36 (ERα36), S1g-2

## Abstract

**Introduction:**

Estrogen receptor (ER) positive patients compromise about 70% of breast cancers. Tamoxifen, an antagonist of ERα66 (the classic ER), is the most effective and the standard first-line drug. However, its efficacy is limited by the development of acquired resistance.

**Methods:**

A specific inhibitor of Hsp70-Bim protein–protein interaction (PPI), S1g-2, together with an inhibitor of Hsp70-Bag3 PPI, MKT-077 and an ATP-competitive inhibitor VER155008, were used as chemical tools. Cell viability assays, co-immunoprecipitation and gene knockdown were used to investigate the role of Hsp70 in tamoxifen resistance. A xenograft model was established in which tamoxifen-resistant breast cancer (MCF-7/TAM-R) cells maintained in the presence of 5 μM tamoxifen were subcutaneously inoculated. The anti-tumor efficiency of S1g-2 was measured after a daily injection of 0.8 mg/kg for 14 days.

**Results:**

It was revealed that Hsp70-Bim PPI protects ERα-positive breast cancer from tamoxifen-induced apoptosis through binding and stabilizing ERα36, rather than ERα66, resulting in sustained EGFR mRNA and protein expression. Disruption of Hsp70-Bim PPI and downregulation of ERα36 expression in tumor samples are consistent with the in vitro functions of S1g-2, resulting in about a three-fold reduction in tumor volume.

**Conclusions:**

The in vivo activity and safety of S1g-2 illustrated that it is a potential strategy for Hsp70-Bim disruption to overcome tamoxifen-resistant ER-positive breast cancer.

**Supplementary Information:**

The online version contains supplementary material available at 10.1186/s13058-024-01790-0.

## Introduction

Breast cancer is a leading cause of cancer-related deaths in women worldwide. About 75% of breast cancers are estrogen receptor (ER) positive [1]. Tamoxifen, an antagonist of ERα66 (the classic estrogen receptor), is the most effective drug and has become the standard first-line therapy for women with ER-positive breast cancer [2]. However, its efficacy is limited by the development of acquired tamoxifen resistance. It has been reported that 20% of all ER-positive breast cancer recur with resistance to tamoxifen, leading to more aggressive neoplasms [3, 4]. Therefore, there is an urgent need to develop new therapeutic approaches that, in combination with tamoxifen, prevent the outgrowth of tamoxifen-resistant breast cancer cells.

In recent years, a large body of clinical and experimental studies have shown that additional cell survival and/or death pathways drive tamoxifen resistance [5, 6]. Overexpression of receptor tyrosine kinases such as epidermal growth factor receptor (EGFR) and the activation of its downstream targets (i.e., AKT, c-Myc, and cyclin D1) serves as an important escape pathway when ERα66 is targeted by tamoxifen [3]. Considerable evidence from experimental studies demonstrates that ERα36, a 36 kDa truncated isoform of ERα66, plays vital roles in promoting breast cancer progressing and in the induction of resistance to tamoxifen treatment [7]. Knocking down ERα36 in tamoxifen-resistant breast cancer cell lines induces downregulation of EGFR both in mRNA level and protein level, by which restoring the sensitivity of ER-positive breast cancer to tamoxifen [8]. Rearrangements that alter ER gene structure and splicing patterns have been described to explain the origin of ERα36 [7]. However, post-translational regulation of ERα36 and the mechanisms of ERα36 proteostasis have not been fully explored.

Heat shock proteins (HSPs) regulate the activity and stability of many oncogenes that control cancer cell survival and progression [9, 10]. A quantitative proteomic study showed that heat shock protein 70 (Hsp70) was the most abundant HSPs identified to associate with ERα [11]. ERα-Hsp70 interaction were detected in the cytoplasm and 17β-estradiol (E2) treatment had no effect on the ERα-Hsp70 interaction [11]. It has been hypothesized that in the absence of estrogenic ligands, ERα is recognized by Hsp40–Hsp70 system followed by its assembly into an Hsp90-based chaperone protein-complex, which keeps this steroid receptor in a ligand-binding competent but inactive state thereby preventing its degradation [12]. However, the experimental evidence showed that unlike the ERα-Hsp90 association that is hormone-dependent, Hsp70 is still associated with ERα in the presence of steroid hormones [11]. These results suggest that Hsp70 plays a different role in regulating ERα compared with Hsp90. Therefore, it would be interesting to explore how the Hsp70 chaperone systems function independently to regulate ERα stability and transcriptional activities.

Hsp70 itself is not a tumor-addict target because of its physiological function [13]. An increasing variety of evidence has shown that heat shock proteins work through protein–protein interactions (PPIs) with diverse co-chaperone, and these PPIs determine the specificity of oncogenic client recognition, resulting in a tumor-specific function. For example, previous studies reported that cochaperones DNAJ, Bag3, Bim, and CHIP can form context-dependent PPIs with Hsp70 to sustain survival in different cancer types [14–16]. Our previous studies identified Hsp70-Bim PPI as a CML-specific target and it protects resistant CML cells through recruitment of specific oncogenic clients [17, 18]. Another PPI pair, Hsp70-Bag3, has been identified to specifically affect tumor invasion pathways in many cancers including breast cancer [19]. As such, Hsp70-Bag3 inhibitor MKT-077 and its analogs are under intensive investigation for breast cancer therapy [20]. However, neither Hsp70-Bim nor Hsp70-Bag3 has been identified to be responsible for ERα proteins and then tamoxifen resistance.

The use of small-molecule inhibitors specifically targeting different PPIs is particularly helpful to reveal the mechanism of tamoxifen resistance because it is controlled primarily by PPIs. A specific inhibitor of Hsp70-Bim PPI, S1g-2 [17], together with MKT-077 [20] and the ATP-competitive inhibitor VER-155008 [21], were chosen. We illustrated that Hsp70-Bim PPI, rather than Hsp70-Bag3 PPI, protects ERα-positive breast cancer from tamoxifen-induced apoptosis through binding and stabilizing more ERα36 than ERα66, resulting in sustained EGFR mRNA and protein expression. The in vivo antagonizing tamoxifen-resistant MCF-7 tumor by S1g-2 illustrated it is a potential strategy for Hsp70-Bim disruption to overcome tamoxifen resistance in ER-positive breast cancer.

## Materials and methods

### Reagents

The compounds tamoxifen, MKT-077, VER-155008, PU-H71 and HCQ were purchased from Selleck Chemicals (Houston, TX, USA). The compound S1g-2 was synthesized according to our previous report [17]. All the chemicals were dissolved in dimethyl sulfoxide (DMSO) to a concentration of 10 mM. To obtain the final concentration, stock solutions were diluted in culture medium. Primary antibodies against LC3 (sc-398822), p62 (sc-28359), β-actin (sc-8432), Bim (sc-374358) were purchased from Santa Cruz Biotechnology (Santa Cruz, CA, USA). A monoclonal antibody raised against a synthetic peptide antigen corresponding to the C-terminal of ERα36 was purchased from Abmart, Inc. (M000803, Shanghai, China). A mouse monoclonal antibody raised against amino acids 2-185 mapping at the N-terminus of ERα66 was purchased from Santa Cruz (sc-8005). To detect both ERα36 and ERα66 on the same gel, a previously reported antibody that can recognize either of ERα36 and ERα66 [22] was used (H222, Research Diagnostic). Antibodies against PARP (ab32138), and Bag3 (ab47124) were purchased from Abcam plc (Cambridge, MA, UK). Antibodies against EGFR (#4267), and Hsp70 (#4873) were purchased from Cell Signaling Technology (Beverly, MA, USA).

### Cell lines

The human breast cancer cell lines MCF-7 and T47D were purchased from American Type Culture Collection (ATCC) and used within 6 months from resuscitation. All cell lines are identified based on short tandem repeat profiles by providers, and mycoplasma contaminations were denied both by providers and at our laboratories. Cells were cultured in DMEM medium (Thermo Scientific HyClone, Beijing, China) for MCF-7 and RPMI-1640 (Thermo Scientific HyClone, Beijing, China) for T47D, supplemented with 10% fetal bovine serum (FBS; Gibco BRL, GrandIsland, NY, USA) and 100 U/mL penicillin–streptomycin and all cells were cultured at 37 °C and 5% CO_2_.

### Generation of tamoxifen-resistant cell lines

MCF-7 or T47D cells were gradually exposed to escalating concentrations of tamoxifen at a rate of 100–200 nM approximately every 10 days from 0.1 to 1 μM, and then a rate of 200–500 nM approximately every 10–20 days from 1 to 5 μM. After an average of 3–6 months of drug escalation, tamoxifen-resistant cells were established from parental MCF-7 and T47D cells. The new clonal cell lines MCF-7/R1, MCF-7/R2 and MCF-7/TAM-R were maintained in the presence of 1 μM, 3 μM and 5 μM tamoxifen, respectively. T47D/TAM-R was maintained in the presence of 5 μM tamoxifen. Before experiments, tamoxifen-resistant cell lines were maintained in a tamoxifen-free medium and passaged at least three times.

### Cell viability assay and analysis of the combination index (CI) values

Viability assessment in cells was performed using CCK-8. Cells (1.0 × 10^4^/well) were cultured and seeded into 96-well plates (three wells per group), and then the cells were treated with a gradient concentration of tamoxifen (0.1–100 µM), S1g-2 (0.1–100 µM), MKT-077 (0.1–100 µM), or VER-155008 (0.1–100 µM) alone or combinations at constant ratios spanning the IC_50_ dose of each agent for 48 h, including MKT-077/tamoxifen combination at 1:5 for MCF-7, 1:10 for MCF-7/TAM-R, 1:5 for T47D, 1:6 for T47D/TAM-R; S1g-2/tamoxifen combination at 2:1 for MCF-7, 1:10 for MCF-7/TAM-R, 2:1 for T47D, 1:8 for T47D/TAM-R; VER-155008/tamoxifen combination at 2:1 for MCF-7, 1:2 for MCF-7/TAM-R, 2:1 for T47D, 1:2 for T47D/TAM-R. DMSO was used at a constant concentration of 0.1% including vehicle-only control wells. Approximately 20 μl of CCK-8 (Dojindo China CO., Ltd) was added to the cells containing 200 μl medium, and the OD value of the cells was measured at 450 nm using a microplate reader (TECAN infinite F200 PRO, Mannedorf, Switzerland) according to the manufacturer’s instructions. The concentration causing 50% cell growth inhibition (IC_50_) was determined from dose response curve using GraphPad Prism software, sigmoidal dose response (Ver. 5.04) (GraphPad Softward, Inc., USA).

Combination index (CI) values were produced by Calcusyn software that utilizes the methodology applied by Chou and Talalay for formal synergy analyses. A CI value of 1.0 indicates an additive effect. CI values less than 1.0 reflect a synergistic effect, whereas CI values greater than 1.0 reflect an antagonistic effect.

### In vivo xenograft tumor model

BALB/c mice (6–8 weeks, female) were purchased from Liaoning Changsheng Biotechnology Co. LTD (LiaoNing, China). A total of 5 × 10^6^ MCF-7/TAM-R cells were subcutaneously inoculated into the right flank of nude mice. Estradiol pellets (0.5 mg per pellet) were implanted s.c. into the right back skin between the ear and shoulder. When the tumors were palpable (~ 100 mm^3^), mice were randomized into two groups (five animals per group). S1g-2 (0.8 mg/kg dose) or vehicle (the ratio of 1:1 dimethyl sulfoxide [DMSO] and phosphate buffer saline [PBS]) was administered intraperitoneal injection daily for 14 days. Neither exclusion criteria nor randomization was performed. The investigator was blinded to the group allocation. Tumor volume and body weight were measured every other day. Tumor volume was determined by using calipers for measurement of the longest (Length) and shortest (Width) dimensions and calculated as (Length × Width^2^)/2. Mice were sacrificed on day 15 and excised tumors were weighed. Excised tumors and organ tissues (liver and Kidney) were cut into blocks, and placed in 10% formalin for paraffin blocks or snap-frozen in liquid nitrogen. For the pathology examination, 4 μm thick tissue sections were stained with hematoxylin & eosin (H&E). For apoptosis assay, tissue terminal deoxynucleotidyl transferase dUTP nick end labelling (TUNEL) assays were performed using ApopTag Plus Peroxidase In Situ Apoptosis Detection kits (Intergen, Purchase, NY, USA) according to the manufacturer’s instructions.

### RT-PCR assays

To assess the transcript level of EGFR, total cellular RNA was isolated using Trizol Reagent (Invitrogen, Carlsbad, CA, USA) according to the manufacturer protocol. The synthesis of the first strand cDNA was performed with Reverse Transcription System using 0.5 µg of RNA and Oligo dT-Adaptor Primer in a 10 μL reaction volume Mixture (TAKARA, Dalian, China). PCR was performed in a 40 μl reaction mixture consisting of 0.25 μl of TaKaRa Ex Taq HS (5 U/μl), 0.5 μL of 20 μM specific primers, 10 μl cDNA, and water as needed. Specific forward and reverse primers (Invitrogen) to produce for optimal amplification of reverse-transcribed cDNA for EGFR were as follows: 5′-CGTCCGCAAGTGTAAGAA-3′ and 5′-AGCAAAAACCCTGTGATT-3′, for β-actin were 5′-TGAGCGCGGCTACAGCTT-3′ and 5′-TCCTTAATGTCACGCACGATTT-3′. The β-actin gene was used as an endogenous control for normalization.

### Generation of Bim knockdown cell lines

The lentiviral vector pLKO.1-EGFP-puro which was a gift from Bob Weinberg (Addgene plasmid # 8453; http://n2t.net/addgene:8453; RRID: Addgene_8453) was used for transfection. Two human Bim siRNAs (#4390824 and #4392420, Invitrogen) were used to transfect breast cancer cell lines using Lipofectamine RANiMAX (Invitrogen) according to the manufacturer’s protocols. For the negative controls, nonspecific shRNA vector was created using a scrambled sequence of the Bim target sequences. Cells were harvested 48 h after transfection.

### Western blotting

Cells were either treated with inhibitor or DMSO (vehicle) and lysed in RIPA buffer (Solarbio, Beijing, China) supplementing Halt protease/phosphatase inhibitor cocktail (Pierce Biotechnology, Rockford, USA) for 30 min on ice and centrifuged at 12,000*g* for 15 min at 4 °C. Protein concentrations were determined using BCA assay (Beyotime, Shanghai, China) according to the manufacturer’s instructions. Lysates (150–250 μg) were electrophoretically resolved by SDS-PAGE, transferred to PVDF membrane and probed with the primary antibodies. Proteins were visualized by the use of Super Signal West Pico Chemiluminescent Substrate (Pierce Biotechnology) and detected on a Kodak Image Station 4000MM Pro (New Haven, CT, USA).

### Co-immunoprecipitation (co-IP)

Cells were washed twice with ice cold PBS and lysed on ice in IP-buffer (10 mM HEPES, pH 7.4, 50 mM NaCl, 5 mM MgCl_2_, 1 mM EGTA, 5% glycerol, 0.5% Triton X-100) supplemented with 1% protease/phosphatase inhibitor for 30 min. After centrifugation (15 min, 12,000*g*, 4 °C), supernatants were collected and normalized to the protein content. 2 μg of antibody were added to an input volume of 200 μL with 5–10 mg/mL protein. After shaking overnight at 4 °C, 20 μl protein G sepharose beads (GE Healthcare, Madison, WI, USA) were added and the samples incubated for 2 h at 4 °C with constant rotation. Immunocomplexes were washed three times with IP buffer and solved by heating for 5 min at 95 °C by western blotting analysis.

To determine the relative levels of Hsp70-Bim PPI and Hsp70-Bag3 PPI, the Bim or Bag3 antibody (Ab) was added at a volume of 10 μl to the lysis buffer and the total volume was adjusted to 200 μl. 20 μl protein G sepharose beads were then incubated with the antibody, and the Ab-bead conjugates were incubated for 2 h at 4 °C. Then, the Ab-bead conjugates was collected by centrifugation, and the supernatant was incubated with Ab and beads again. After five successive precipitations, the 5 collections of Ab-bead conjugates mix together and were washed three times with lysis buffer and separated by SDS-PAGE, followed by a standard western blotting procedure.

### Bioinformatic analysis of clinical data

Genetic differential analysis of 1085 breast tumor mesenchymal samples and 291 normal breast mesenchymal samples was performed using the GEPIA2 database. The results of the genetic differential analysis of GEPIA2 were compared using two databases, TCGA and GTEx, and the tumor tissue from TCGA was used to compare with the normal tissue from GTEx. Patient survival curves were obtained using DrugSURV by analyzing the GEO dataset ID GSE24450 that contains “183 breast tumors from the Helsinki University center hospital with survival information”. HSPA1A (ID 3303, probe ID: ILMN_1789074) levels were defined compared to the median Hsp70 expression in all patients.

### Statistics

Data are presented as mean ± standard deviation (SD) from three independent experiments, and were compared using the one-way ANOVA test. All of the experiments were performed at least in triplicate. Sample sizes are indicated in figure legends and selected to provide > 80% power.

## Results

### S1g-2 is more effective in tamoxifen-resistant breast cancer cell lines than their sensitive counterparts while MKT-077 and VER-155008 aren’t

**Fig. 1:**
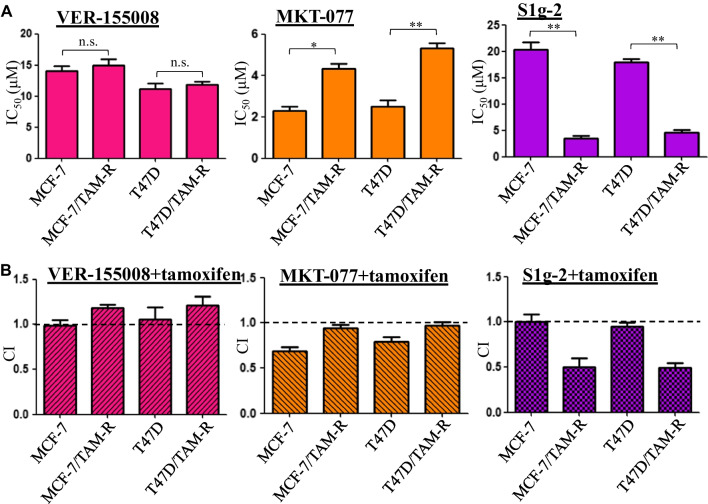
S1g-2 is more effective in tamoxifen-resistant breast cancer cell lines than their sensitive counterparts **A** IC_50_ values for VER-155008, MKT-077, and S1g-2 at 48 h in MCF-7, MCF-7/TAM-R, T47D and T47D/TAM-R cells, determined by the CCK-8 assay. The data are expressed as mean ± SD (n = 3 biologically independent experiments) *P < 0.05, **P < 0.01 (one-way ANOVA test); n.s. indicates no significance. **B** Combination index (CI) values of, tamoxifen/VER-155008, tamoxifen/MKT-077, and tamoxifen/S1g-2 combination respectively in MCF-7, MCF-7/TAM-R, T47D and T47D/TAM-R cells

ER-positive breast cancer cell lines MCF-7 and T47D were acclimatized to grow in the medium containing 5 μM of tamoxifen, yielding MCF-7/TAM-R and T47D/TAM-R respectively. The lethality of three different Hsp70 inhibitors and their potential to synergize with tamoxifen were measured by CCK-8 assay. Firstly, a 3–fourfold higher IC_50_ of tamoxifen in MCF-7/TAM-R and T47D/TAM-R cells than their parental counterparts were detected, confirming stable resistance to tamoxifen (Additional file 2: Fig. S1 and Table S1). Then, the ATP-competitive Hsp70 inhibitor VER-155008 showed equally low killing ability either in the tamoxifen-sensitive or tamoxifen-resistant cell lines (Fig. 1A and Additional file 2: Fig. S1), even though the Hsp70 expression was elevated about twofold in tamoxifen-resistant cell lines than the sensitive counterparts (Additional file 2: Fig. S2). No synergism was found between VER-155008 and tamoxifen (Fig. 1B and Additional file 2: Table S2). It challenges the contribution of Hsp70’s level to tamoxifen-resistant breast cancer therapy.

However, the bioinformatic analysis of Hsp70 expression from the public database showed a much higher Hsp70 expression in invasive breast cancers than in healthy breast tissues (*P* = 0.012, Additional file 2: Fig. S3A). Additionally, breast cancer patients with higher Hsp70 expression have poorer survival compared to patients with low Hsp70 expression (*P* = 0.015, Additional file 2: Fig. S3B). We then investigated if the protein–protein interactions (PPIs) involved Hsp70 protein contribute to tamoxifen resistance by using a Hsp70-BAG3 inhibitor MKT-077 and a Hsp70-Bim inhibitor S1g-2. Not surprisingly, MKT-077 displayed less sensitivity in MCF-7/TAM-R and T47D/TAM-R cells than in MCF-7 and T47D cells (IC_50_ values of 4.5 and 5.6 μM, vs 2.1 μM and 2.5 μM, respectively) (Fig. 1A and Additional file 2: Table S1). In contrast, although S1g-2 displayed poor IC_50_ values in MCF-7 and T47D (20.3 μM and 18.3 μM respectively), it exhibited 4–sixfold higher killing potency in tamoxifen-resistant breast cancer cell lines (IC_50_ = 3.5 μM and 4.6 μM, respectively in MCF-7/TAM-R and T47D/TAM-R) (Fig. 1A and Additional file 2: Table S1). The IC_50_ values are comparable with those in CML cells reported by us [17].

Next, the synergy of MKT-077 with tamoxifen and S1g-2 with tamoxifen also showed opposite trends (Fig. 1B and Additional file 2: Table S2). MKT-077 exhibited synergy with tamoxifen in the parental MCF-7 and T47D (CI = 0.6 and 0.7, respectively) but no synergy in resistant cell lines. S1g-2 exhibited a highly synergistic effect (CI = 0.4 and 0.5, respectively) in the resistant cells but not in parental cell lines. It suggested that S1g-2 could overcome tamoxifen resistance, alone or in combination with tamoxifen. However, MKT-077 could not. The consistent synergy of tamoxifen and S1g-2 by Annexin V staining further supported it (Additional file 2: Fig. S4).

### Hsp70-Bim PPI, instead of Bsp70-Bag3, provides increased protection of breast cancer cells against apoptosis along with increased levels of tamoxifen resistance

**Fig. 2:**
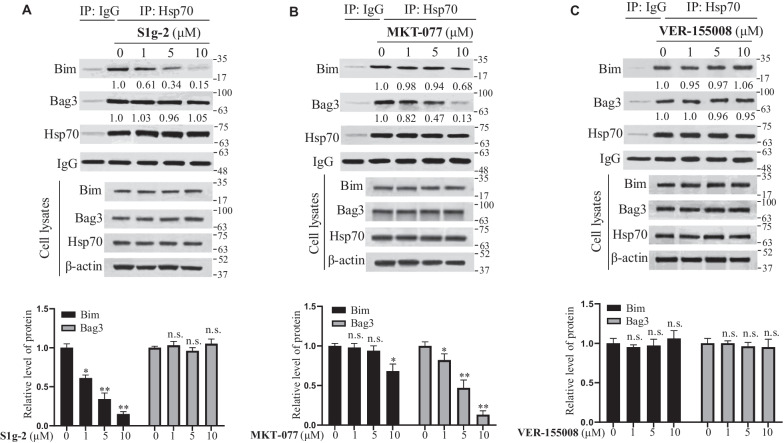
S1g-2 selectively disrupted Hsp70-Bim PPI, while MKT-077 preferentially disrupted Hsp70-Bag3 PPI. Western blot and co-IP analysis of the levels of Hsp70, Bim, Bag3, and their PPIs in MCF-7/TAM-R cells upon treatment with a gradient of concentrations (0–10 μM) of S1g-2 (**A**), MKT-077 (**B**), and VER-155008 (**C**) for 12 h, respectively, using β-actin as a loading control. An equivalent of DMSO were added to the compound untreated group as vehicle control. The graphs show (mean ± SD, n = 3 biologically independent experiments) Bim or Bag3 protein level in Hsp70 co-IP normalized against β-actin and relative to that in control. *P < 0.05, **P < 0.01 (one-way ANOVA test); n.s. indicates no significance between vehicle- and compounds-treated groups

The effect of MKT-077 and S1g-2 on their individual target, Hsp70-Bag3 PPI and Hsp70-Bim PPI, were evaluated in situ respectively. ATP-competitive Hsp70 inhibitor VER-155008 was assayed in parallel as a negative control. By co-immunoprecipitation (co-IP) experiments, dose-dependent disruption of the Hsp70-Bim PPI in MCF-7/TAM-R was found for S1g-2 at the concentration range of 1–10 μM, while the Hsp70-Bag3 PPI showed no change upon the treatment (Fig. 2A). MKT-077 failed to inhibit Hsp70-Bim PPI at 5 μM, and only a few disruptions occurred at a high concentration of 10 μM which is twice of its IC_50_ in this cell line. MKT-077 exhibited much higher potency to disrupt the Hsp70-Bag3 PPI, consistent with the previous report [20] (Fig. 2B). VER-155008 failed to disrupt both Hsp70-Bim and Hsp70-Bag3 PPI (Fig. 2C). The results confirmed the on-target activity of the two PPI inhibitors in breast cancer cell lines, and suggested that Hsp70-Bim PPI, rather than Hsp70-Bag3 PPI or Hsp70 itself, protects MCF-7/TAM-R and T47D/TAM-R.

**Fig. 3:**
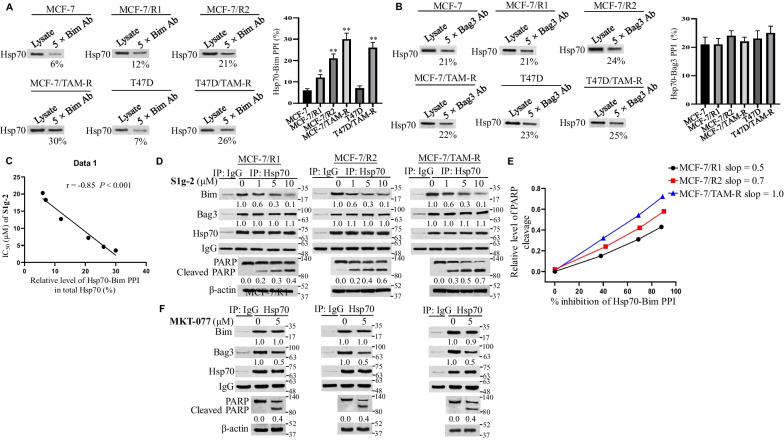
Hsp70-Bim PPI, instead of Hsp70-Bag3 PPI, confers increased level of tamoxifen resistance. Hsp70 from cell lysates was isolated through sequential immunoprecipitation steps using Bim antibody (**A**) or Bag3 antibody (**B**), followed by western blot using Hsp70 antibody. The data underneath the bands were calculated by the proportion of relative Hsp70 level pulled down by 5 × Bim or 5 × Bag3 antibody to that in cell lysates. The graphs in the right panel show (mean ± SD, n = 3 biologically independent experiments) relative levels of Hsp70-Bim PPI and Hsp70-Bag3 PPI. *P < 0.05, **P < 0.01 (one-way ANOVA test); n.s. indicates no significance. **C** Correlation of the relative levels of Hsp70-Bim PPI in different cell lines with the IC_50_ values of S1g-2. **D** Co-IP and western blot analysis of the relative levels of Hsp70-Bim PPI, Hsp70-Bag3 PPI and PARP cleavage in MCF-7/R1, MCF-7/R2 and MCF-7/TAM-R with a gradient of concentrations (0–10 μM) of S1g-2 treatment for 12 h (co-IP assay) and 24 h (PARP cleavage assay), respectively. **E** Correlation of the percentage of S1g-2-induced Hsp70-Bim PPI inhibition with the relative level of PARP cleavage. **F** Co-IP and western blot analysis of the relative levels of Hsp70-Bim PPI, Hsp70-Bag3 PPI and PARP cleavage in MCF-7/R1, MCF-7/R2 and MCF-7/TAM-R without or with 5 μM MKT-077 treatment for 12 h (co-IP assay) and 24 h (PARP cleavage assay), respectively. All figures represent the results from n = 3 biologically independent experiments

To test the association of Hsp70-Bim PPI with the survival of tamoxifen-resistant breast cancer cells, gradient tamoxifen-resistant MCF-7 cell lines were established that adapted for growth in the presence of 1 μM and 3 μM tamoxifen, respectively and named as MCF-7/R1 and MCF-7/R2. They were tested together with MCF-7/TAM-R which is adapted to 5 μM tamoxifen. Their IC_50_ values for tamoxifen are 21.4 (MCF-7/R1), 32.6 μM (MCF-7/R2) and 44.5 μM (MCF-7/TAM-R) (Additional file 2: Table S1). Then, we performed co-IPs with Bim or Bag3 antibodies to quantify the relative level of the Hsp70-Bim PPI and Hsp70-Bag3 PPI respectively across the panel of cell lines. As shown in Fig. 3A, the percentage of Hsp70 pulled down by the Bim antibody increased along with the increased level of resistance from parental MCF-7 to MCF-7/R1, MCF-7/R2 and MCF-7/TAM-R (6%, 12%, 21% and 30%). A similar increase was found in T47D/TAM-R compared with T47D (26% versus 7%) (Fig. 3A). In contrast, there was no significant change in Hsp70-Bag3 PPI (Fig. 3B). The relative level of the Hsp70-Bim PPI inversely correlated with the IC_50_ of S1g-2 (Fig. 3C). These data illustrated that in the order of MCF-7/R1, MCF-7/R2 and MCF-7/TAM-R, Hsp70 formed more and more PPIs with Bim. The data confirmed that Hsp70-Bim PPI, rather than Hsp70-Bag3 PPI, protects breast cancer cells from tamoxifen killing. It is the mechanism under which S1g-2 overcomes tamoxifen resistance.

Next, we selected the time point of 12 h for Hsp70 co-IP to avoid the interference of apoptosis, and western blot on PARP cleavage at 24 h according to a time course study (Additional file 2: Fig. S5). As shown in Fig. 3D, in MCF-7/R1, MCF-7/R2 and MCF-7/TAM-R, S1g-2 treatment resulted in consistent dose-dependent inhibition of Hsp70-Bim PPI. Importantly, apoptosis contributed by the average percentage of Hsp70-Bim PPI disruption is increased along with the increased level of resistance, as shown by the increased slope between the percentage of Hsp70-Bim PPI disruption and PARP cleavage in the order of MCF-7/R1, MCF-7/R2 and MCF-7/TAM-R (0.5, 0.7, 1.0, respectively) (Fig. 3E). In contrast, 5 μM MKT-077 was unable to disrupt Hsp70-Bim PPI and did not exhibit enhanced apoptosis in tamoxifen-resistant cell lines, despite more than 50% disruption of Hsp70-Bag3 PPI occurred (Fig. 3F).

### Hsp70-Bim PPI selects for ERα36 instead of ERα66

**Fig. 4:**
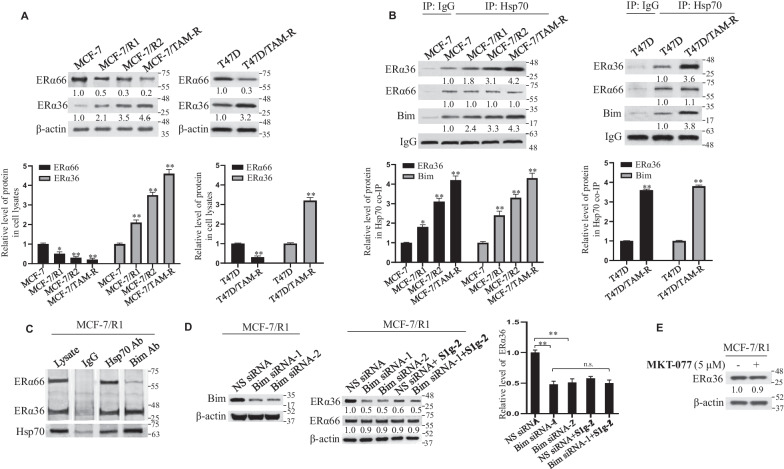
Hsp70-Bim PPI preferentially binds and stabilizes ERα36 instead of ERα66. **A** Western blot analysis of the relative levels of ERα36 and ERα66 using respectively specific monoclonal antibody in parental tamoxifen-sensitive MCF-7 and tamoxifen-resistant cell lines MCF-7/R1, MCF-7/R2 and MCF-7/TAM-R (left panel), as well as parental T47D and tamoxifen-resistant cell line T47D/TAM-R (right panel), using β-actin as a loading control. Bottom: relative level of ERα36 and ERα66. The data are expressed as mean ± SD (n = 3 biologically independent experiments) *P < 0.05, **P < 0.01 (one-way ANOVA test). **B** Co-IP analysis of Hsp70 interactions with ERα36 and Bim in tamoxifen-sensitive and resistant MCF-7 cell lines (left panel) and T47D cell lines (right panel). Bottom: relative level of protein in Hsp70 co-IP. The data are expressed as mean ± SD (n = 3 biologically independent experiments). *P < 0.05, **P < 0.01 (one-way ANOVA test). **C** Western blot of Hsp70 complexes using an H222 antibody raised against an epitope common to ERα36 and ERα66 in MCF-7/R1 extracts isolated by precipitation with a nonspecific IgG, Hsp70 antibody or Bim antibody. **D** Western blot analysis of the levels of ERα36 and Bim using specific monoclonal antibody in non-specific (NS) siRNA—and 2 independent human Bim-siRNA transfected MCF-7/TAM-R cells with vehicle control or S1g-2 (5 μM) treatment for 12 h. Right: the relative level of ERα36. The data are expressed as mean ± SD (n = 3 biologically independent experiments). **P < 0.01 (one-way ANOVA test); n.s. indicates no significance. **E** Western blot analysis of the levels of ERα36 in MCF-7/R1 upon treatment with vehicle control or MKT-077 (5 μM) for 12 h, using β-actin as a loading control

ERα proteins are well-known Hsp70 clients [23]. A recent quantitative proteomic study showed that heat shock protein 70 (Hsp70) is the most abundant heat shock protein identified to associate with ERα [11]. As such, we investigated if the increasing amounts of PPIs in resistant cells could distinguish the two isoforms of ERα. Two monoclonal antibodies, one raised against the unique C-terminal of ERα36 [8] and the other raised against the unique N-terminus of ERα66, were used to specifically recognize the two ERα isoforms. As shown in Fig. 4A, we detected progressively increased levels of ERα36 but decreased levels of ERα66 in MCF-7/R1, MCF-7/R2, MCF-7/TAM-R compared to parental MCF-7, consistent with the previous reports [8]. Compared to T47D, a significant downregulation of ERα66 accompanied with upregulation of ERα36 were also detected in T47D/TAM-R cells (Fig. 4A, right panel). Co-IP of Hsp70 showed that accompanied with progressively increased Hsp70-Bim PPI, Hsp70 binds increasing amounts of ERα36 in the order of MCF-7/R1, MCF-7/R2 and MCF-7/TAM-R (Fig. 4B, left panel), as well as in T47D/TAM-R cells (Fig. 4B, right panel) compared to their parental cell lines. ERα66 binds with Hsp70 at a constant level among all the cell lines (Fig. 4B). The data suggested that Bim helps Hsp70 to bind and stabilize ERα36 instead of ERα66.

To test it, we performed immunoprecipitations with Bim antibody and analyzed the associated ERα species in MCF-7/R1 because this cell line expresses comparable amounts of ERα66 and ERα36. These two ERα species are clearly separable by molecular weight and are therefore easily distinguishable by western blot with a H222 antibody raised against an epitope common to ERα36 and ERα66 [22]. As shown in Fig. 4C, Bim antibody, but not a nonspecific IgG, isolated Hsp70 complexed with a large amount of ERα36 but a small amount of ERα66, while the Hsp70 antibody pulled down comparable amounts of ERα66 and ERα36. It suggested that the subset of Hsp70 bound to Bim, but not the total Hsp70, selects for ERα36 instead of ERα66.

Further, we tested the effect of Bim silence on the level of ERα36 in MCF-7/R1 by western blot using ERα36-specific monoclonal antibody. As shown in Fig. 4D, levels of ERα36 were decreased by about 50% in MCF-7/R1 cells transfected with two independent Bim siRNAs compared with control siRNA treatment, while ERα66 levels were decreased by 10%. Given that S1g-2 and Bim competitively bind to the same pool of Hsp70 [17], we used S1g-2 to examine the specificity of Hsp70-Bim for ERα36 over ERα66. As shown in Fig. 4D, ERα36 was significantly more susceptible to degradation by S1g-2 than was ERα66.

In contrast, MKT-077 had little effect on ERα36 just as it cannot affect Hsp70-Bim PPI (Fig. 4E). These data showed that the dependence of ERα36 on Hsp70 is facilitated by Bim rather than Bag3.

These data strongly supported that Hsp70 use and require more acutely the cochaperone Bim when it modulates the activity of ERα36 but not ERα66.

### S1g-2 overcomes tamoxifen resistance through disruption of Hsp70/Bim/ERα36 complex

The similar downregulation of ERα36 by S1g-2 and Bim silence is unveiling the killing function of S1g-2 (Fig. 4D). Further, in cells with stable Bim knockdown, S1g-2 treatment had little effect on the levels of ERα36 (Fig. 4D, right panel), illustrating S1g-2 exhibits killing functions through Hsp70/Bim/ERα36 complex.

**Fig. 5:**
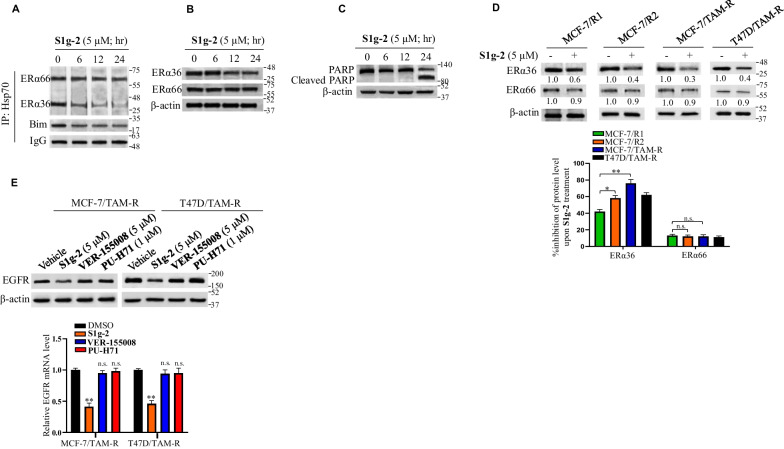
S1g-2 kills tamoxifen-resistant cells through Hsp70/Bim/ERα36 mediated signaling pathway. **A** Western blot of Hsp70 complexes using an H222 antibody that can recognize both ERα66 and ERα36, as well as Bim antibody in MCF-7/R1 cells upon treatment with 5 μM S1g-2 treatment for 0, 6, 12 or 24 h. **B, C** Western blot analysis of the levels of ERα66 and ERα36 using respectively specific monoclonal antibody, as well as PARP cleavage in MCF-7/R1 cells upon treatment with 5 μM S1g-2 treatment for 0, 6, 12 or 24 h. **D** Western blot analysis of the levels of ERα36 and ERα66 using respectively specific monoclonal antibody in MCF-7/R1, MCF-7/R2, MCF-7/TAM-R and T47D/TAM-R cells upon treatment with S1g-2 (5 μM) for 12 h, using β-actin as a loading control. An equivalent of DMSO was added to the compound untreated group as vehicle control. Bottom: the percentage inhibition of ERα36 and ERα66 upon S1g-2 treatment. The data are expressed as mean ± SD (n = 3 biologically independent experiments). *P < 0.05, **P < 0.01 (one-way ANOVA test); n.s. indicates no significance. **E** Western blot analysis of the levels of EGFR in MCF-7/TAM-R and T47D/TAM-R cells upon treatment with vehicle control, S1g-2 (5 μM), VER-155008 (5 μM) and PU-H71 (1 μM) for 12 h, using β-actin as a loading control. Bottom: Relative mRNA level of EGFR determined by real-time qPCR. β-actin gene was used as an endogenous control for normalization. The data are expressed as mean ± SD (n = 3 biologically independent experiments). *P < 0.05, **P < 0.01 (one-way ANOVA test)

To examine when ERα36 decreases and apoptosis initiates upon Hsp70/Bim/ERα36 complex disruption by S1g-2, we performed experiments on time course of Hsp70 co-IP, western blot for ERα36, and PARP cleavage in MCF-7/R1 cells. As shown in Fig. 5A, the amount of ERα36, but not ERα66, in Hsp70 complex was significantly decreased by S1g-2 at 6 h, accompanied with the decrease of Bim in Hsp70 complex. ERα36 was downregulated at 12 h (Fig. 5B), and PARP cleavage was detected at 24 h (Fig. 5C). The data showed that S1g-2 induces ERα36 degradation following disruption of Hsp70/Bim/ERα36, after which apoptosis was triggered. These data identified that S1g-2 kills tamoxifen-resistant cells through Hsp70/Bim/ERα36 mediated signaling pathway. Western blot analysis of ERα36 in a series of tamoxifen-resistant cell lines upon S1g-2 treatment at 12 h showed that in accord with the progressively increased Hsp70/Bim/ERα36 complex, the downregulated degree of ERα36 enhanced in the order of MCF-7/R1, MCF-7/R2 and MCF-7/TAM-R (40%, 60% and 70%) (Fig. 5D). Similarly, 60% downregulation of ERα36 was observed in T47D/TAM-R cells. No such enhancement was observed for ERα66 downregulation (Fig. 5D), further illustrating ERα36, rather than ERα66, dependents on Hsp70-Bim PPI.

As the transcriptional target of ERα36, EGFR exhibited downregulation at both mRNA level and protein level upon S1g-2 treatment, but not ATP-competitive Hsp70 inhibitor VER-155008 as well as Hsp90 inhibitor PU-H71 in either MCF-7/TAM-R cells or T47D/TAM-R cells (Fig. 5E). The results showed that Hsp70-Bim PPI not only stabilizes ERα36 level but also facilitates its traffic into nuclei and then acts on oncogenic target genes.

### S1g-2 inhibits tamoxifen-resistant tumor growth in vivo

**Fig. 6:**
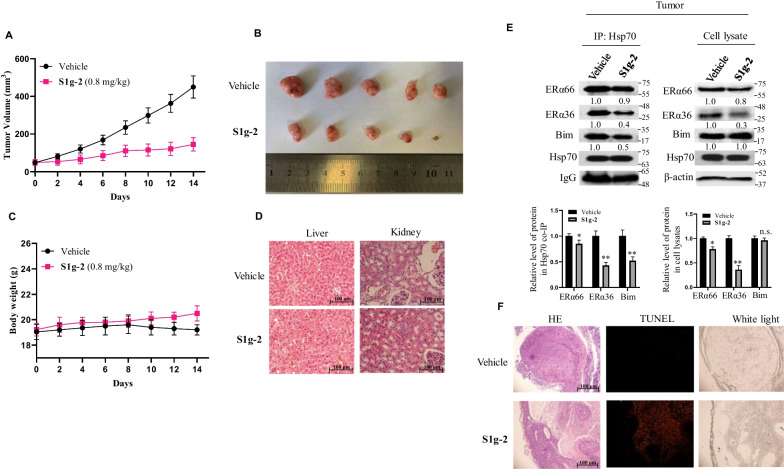
In vivo efficacy of S1g-2 in xenograft models of MCF-7/TAM-R. **A** Tumor volume curve after treatment with 0.8 mg/kg S1g-2. The data are plotted as the means ± SEM (n = 5 mice for each group). **B** Tumors isolated from vehicle- and S1g-2-treated groups (n = 5), respectively, at the end of treatment. **C** The mouse body weight during the treatment period. The data are plotted as the means ± SEM (n = 5 mice for each group). **D** Representative H&E stainning of liver and Kidney tissues from vehicle- and S1g-2-treated groups (n = 5), respectively. **E** Western blot and co-IP analysis of the xenograft tumors derived from vehicle- and S1g-2-treated groups. The graphs show (mean ± SD, n = 3 biologically independent experiments) Bim, ERα36 and ERα66 protein level in Hsp70 co-IP and cell lysates in S1g-2-treated groups normalized against β-actin and relative to that in vehicle-treated groups. *P < 0.05, **P < 0.01 (one-way ANOVA test); n.s. indicates no significance between vehicle- and S1g-2-treated groups. **F** Tumors were sectioned and subsequently stained with H&E (left panels) or TUNEL (middle panels) with white light imaging (right panels)

To test the potential therapeutic effects of S1g-2 in vivo, we established a xenograft model in which MCF-7/TAM-R cells were injected subcutaneously into nude mice, and then treated with S1g-2 (0.8 mg/kg dose) daily for 14 days. As shown in Fig. 6A, S1g-2 treatment led to significantly reduced tumor volume compared to the vehicle alone (control group). At the end of treatment, S1g-2 group had an average three-fold reduction in tumor volume compared to the control (Fig. 6B). Moreover, this inhibition showed no obvious toxicity, as body weight and hematoxylin and eosin (H&E)-stained sections of major organs did not greatly change (Fig. 6 C and D).

The effect of S1g-2 on Hsp70-Bim PPI and the level of ERα in MCF-7/TAM-R xenografts were examined by co-IP and western blotting analysis. As shown in Fig. 6E, administration of S1g-2 led to significant disruption of Hsp70-Bim PPI accompanied by significantly downregulated ERα36 expression in tumor samples, confirming that S1g-2 inhibited Hsp70-Bim in vivo as demonstrated previously in vitro experiments. Furthermore, apoptotic cell death was examined by a TUNEL assay in paraffin-embedded tumor sections. As shown in Fig. 6F, S1g-2 treatment significantly increased the TUNEL-positive apoptotic cell population compared to those in the vehicle-treated control.

## Discussion

**Fig. 7:**
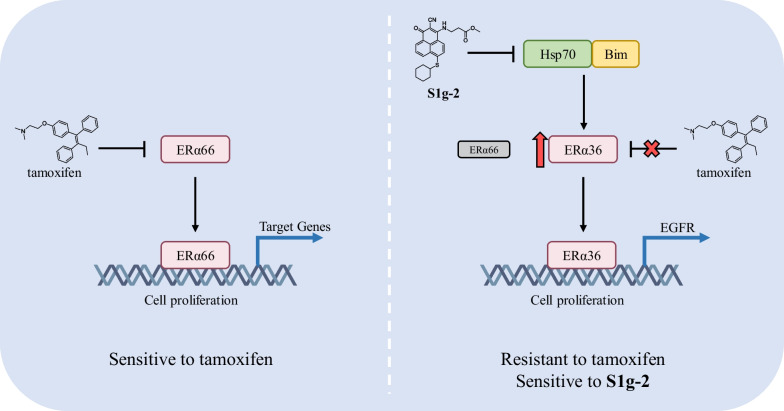
Graphic summary of our work

Multichaperone complexes formed with different cochaperones through PPIs always exhibit distinct functions. The particular role of Hsp70-Bim PPI in overcoming tamoxifen resistance of ER-positive breast cancer was unveiled for the first time. As shown in Fig. 7, this chaperone pair contributes to tamoxifen resistance through binding and stabilizing oncogenic client ERα36 over ERα66, which in turn activates EGFR mRNA and protein expression. Hso70-Bim PPI exhibited a unique role which is distinguished from Hsp70-Bag PPI and independent of Hsp90.

The mechanism can explain the higher cell-killing activity of S1g-2 in tamoxifen-resistant breast cancer cell lines than that in parental tamoxifen-sensitive cell lines. ERα36 is overexpressed upon tamoxifen exposure and inhibits ERα66, as previously reported by other groups and detected by us here. It means that tumor addiction switches from ERα66 to ERα36. Hsp70 proteins bind both ERα36 and ERα66, as showed by previous proteomic study [11] and detected here. However, Bim, rather than Bag3, helps Hsp70 to bind and stabilize ERα36 over ERα66 as shown by the comparison of S1g-2 and MKT-077, and supported by Bim silencing experiments. Most likely it is not only due to the increased expression of ERα36 seen with tamoxifen resistance but the relatively higher affinity of ERα36 toward Hsp70, because in the MCF-7/R1 cells, Hsp70-Bim PPI binds much more ERα36 than ERα66 even their expression level is similar.

The selectivity of binding could be attributed to the conformational change of Hsp70 upon Bim engagement given on that Bim binds in an allosteric site of Hsp70 [24]. Not all the Hsp70, but a subset that binds Bim, could interact with ERα36. It could be explained that Bim allosterically induces a specific conformation of Hsp70 to facilitate preferential ERα36 binding. Consistently, disruption of Hsp70-Bim also prefers to dissociate Hsp70 interaction with ERα36 over ERα66. Together with what we found in CML that Bim binds to a pool of Hsp70, which accounts for only a small part of total Hsp70, but it recruits a specific group of CML addicted clients, we identified conformational-dependent Hsp70/Bim/clients complex. These results add to, and clarify, previous reports detailing a new series of clients of Hsp70-Bim PPI. Our study supported the concept that different chaperone pair by diverse PPIs recruit different kinds of clients, and the same tumors would change their addiction to a particular chaperone pair due to the environmental change.

Moreover, Hsp70 not only stabilizes ERα36 but maintain its cytoplasmic-nuclear trafficking with the help of Bim because we found its transcriptional target changing upon S1g-2 exposure. It is distinguished with Hsp90 since Hsp90 inhibitor PU-H71 had no significant effect on ERα36’s transcriptional activity. It means that Hsp70 binds with an active conformation of ERα36, or the binding site does not affect DNA binding activity of ERα36. It is consistent with the previous finding that Hsp70 keeps ERα36 in the active states in transcription-one is “on-site” (associated with chromatin) [25] while Hsp90 complexes with non-active status of ERα36, and one is “off-site” (not associated with chromatin) [26, 27]. We have illustrated the independent role of Hsp70-Bim compared with Hsp90 in CML. These results add to, and clarify, previous reports detailing a new unique function of Hsp70-Bim PPI. This study demonstrated that Hsp70-involved PPIs are appealing tumor targets instead of Hsp70 itself. As such, Hsp70-Bim PPI represents a very attractive target for advancing breast cancer treatment.

The stronger killing effect of S1g-2 against tamoxifen-resistant breast cancer cell lines in cell-based experiments and in vivo differ it from all known Hsp70 inhibitors, endowing it as a first-in-class promising lead compounds. This has important implications for the development of Hsp70 inhibitor drugs. Although several Hsp70 inhibitors have entered the clinical phase, their clinical application is limited by toxicity toward normal cells, and none of them have been clinically approved by the US Food and Drug Association [28–30]. Since cancer-specific functions of Hsp70 are found closely associate with Hsp70 PPIs, specific inhibitors targeting cancer-related Hsp70-involved PPIs were quickly developed, including JG-98/JG-231 [20, 31], MAL3-101 [32], apoptozole [33] and AEAC [34]. S1g-2 has added a new content in this area.

### Supplementary Information

**Additional file 1**. Original western blot.

**Additional file 2**. Supplementary Figures and Tables.

## Data Availability

All data generated or analyzed during this study are included in this published article.
