# Supplementary material for: Small molecule inhibitor targeting the Hsp70-Bim protein–protein interaction in estrogen receptor-positive breast cancer overcomes tamoxifen resistance
Source: Breast Cancer Res. 2024 Feb 26;26:33. doi: 10.1186/s13058-024-01790-0 (PMC10895875; doi:10.1186/s13058-024-01790-0)

Fig. 2A

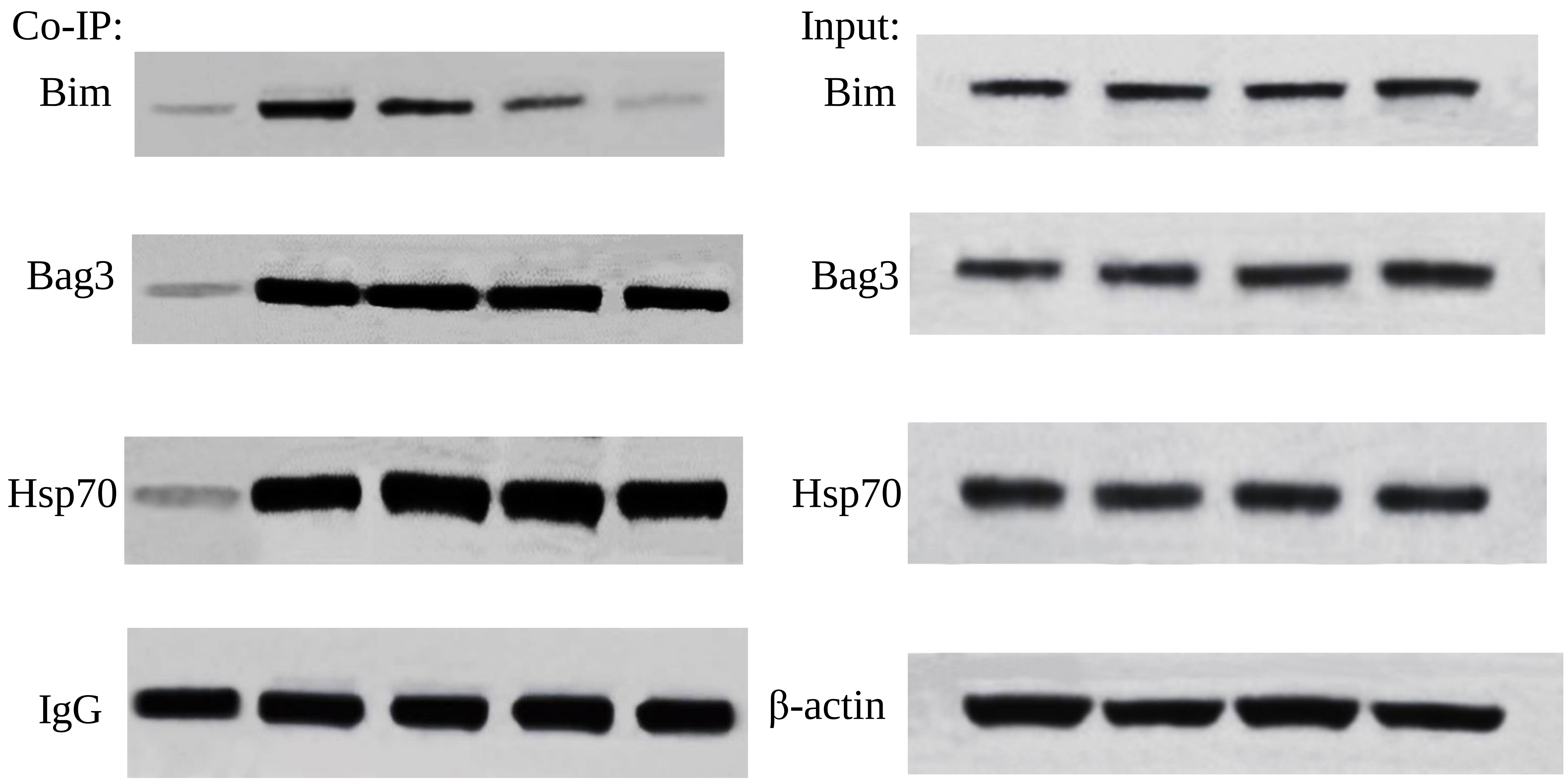

Fig. 2B

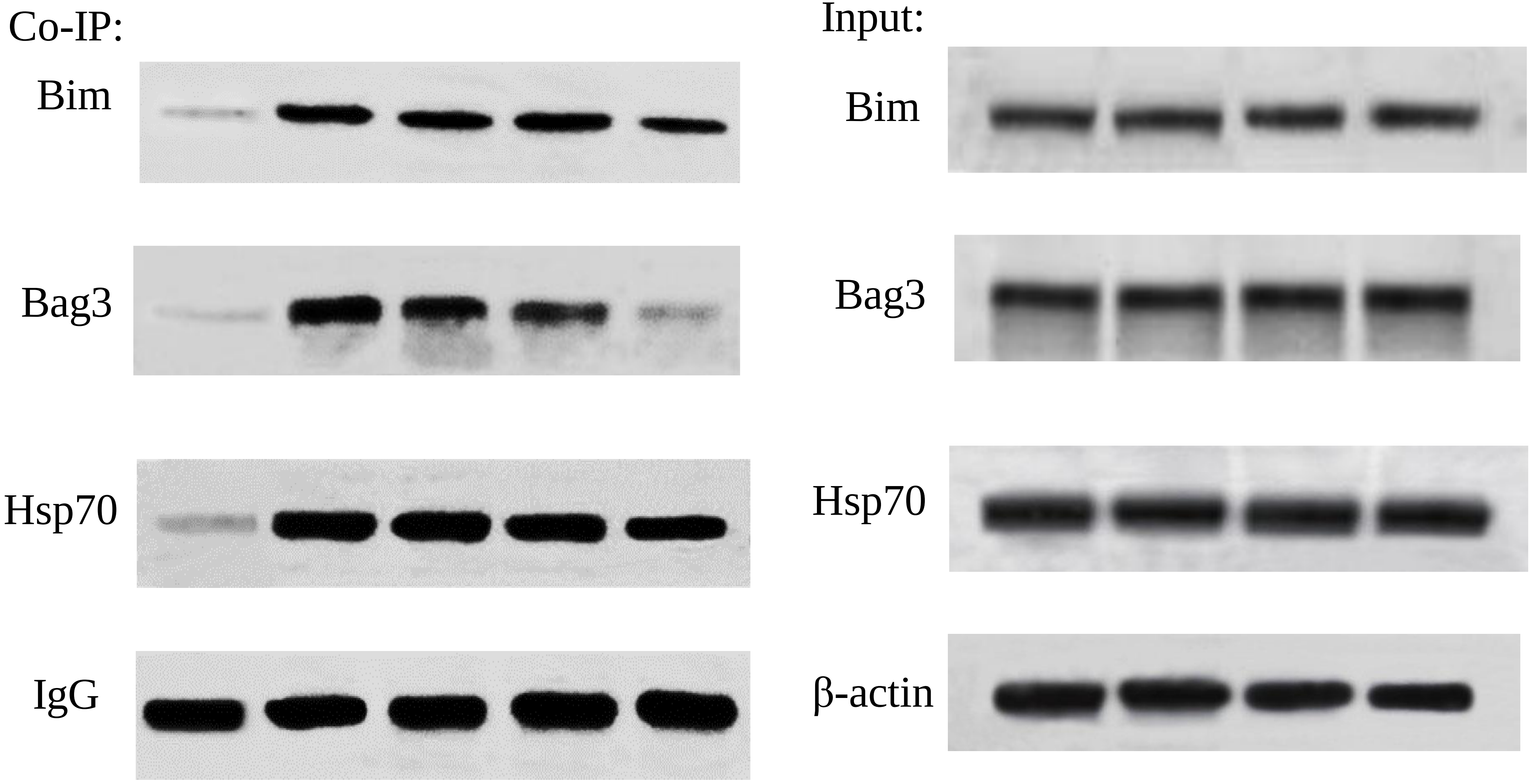

Fig. 2C

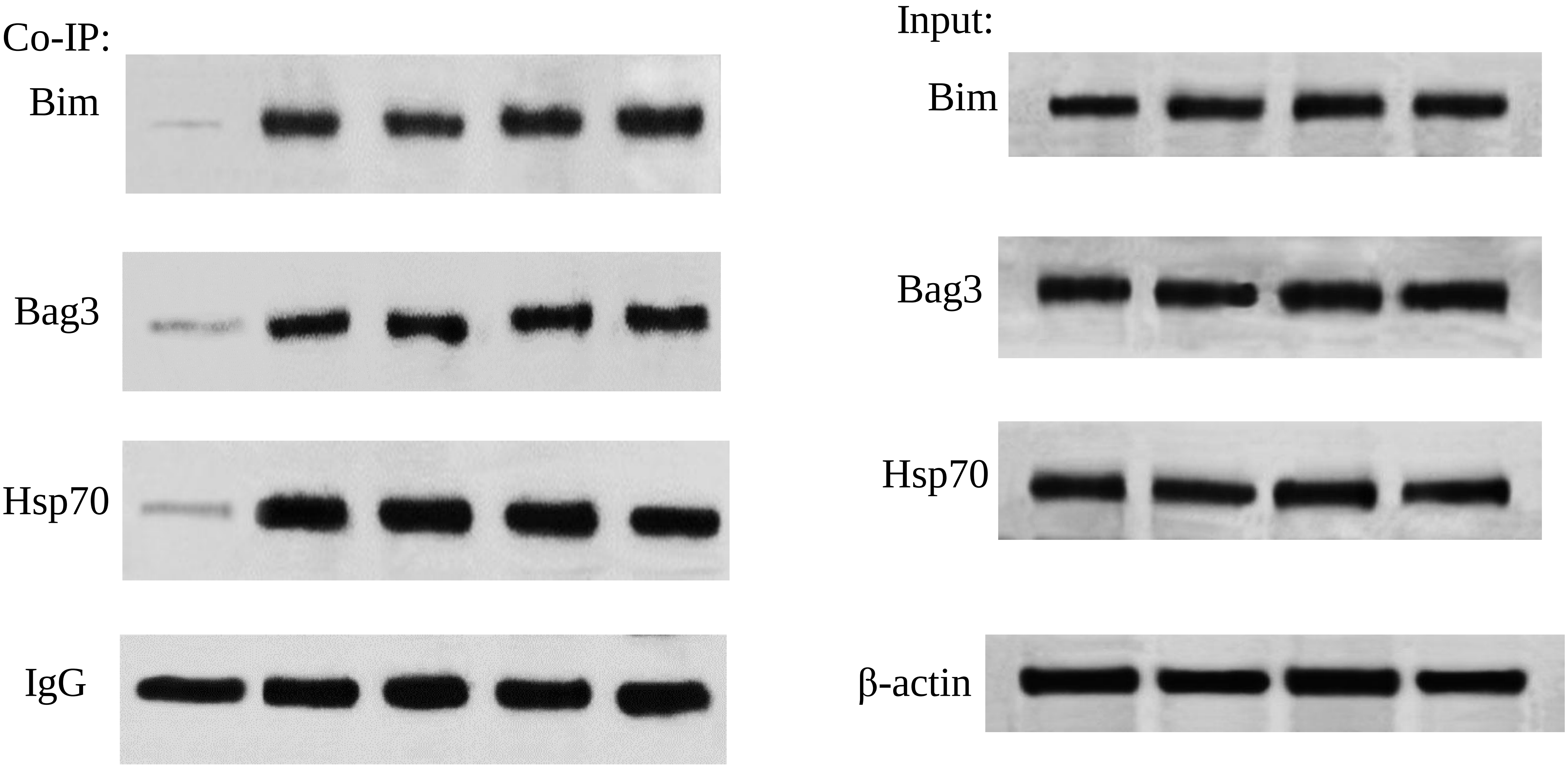

Fig. 3A

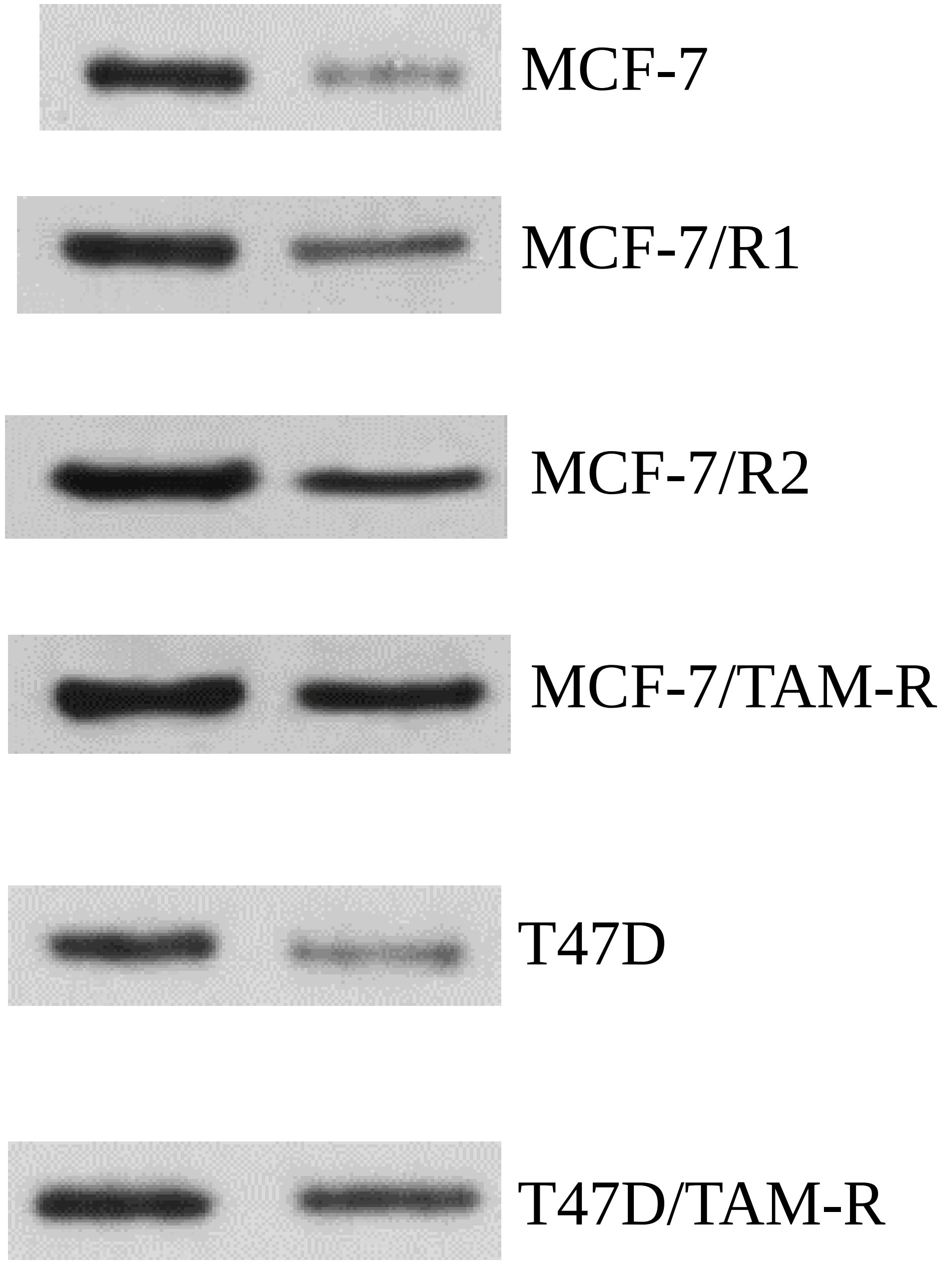
MCF-7
MCF-7/R1
MCF-7/R2
MCF-7/TAM-R
T47D
T47D/TAM-R

Fig. 3B

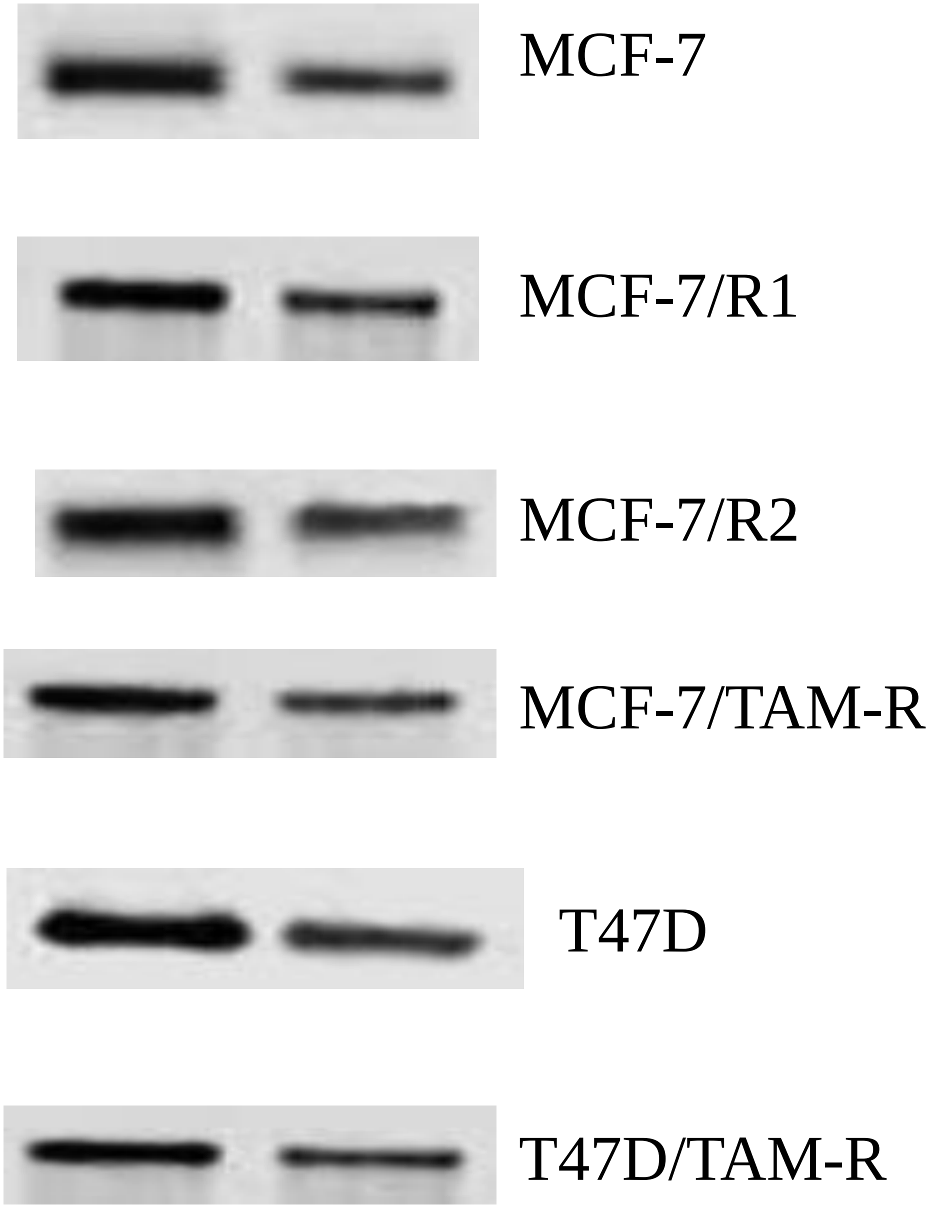
MCF-7
MCF-7/R1
MCF-7/R2
MCF-7/TAM-R
T47D
T47D/TAM-R

Fig. 3D

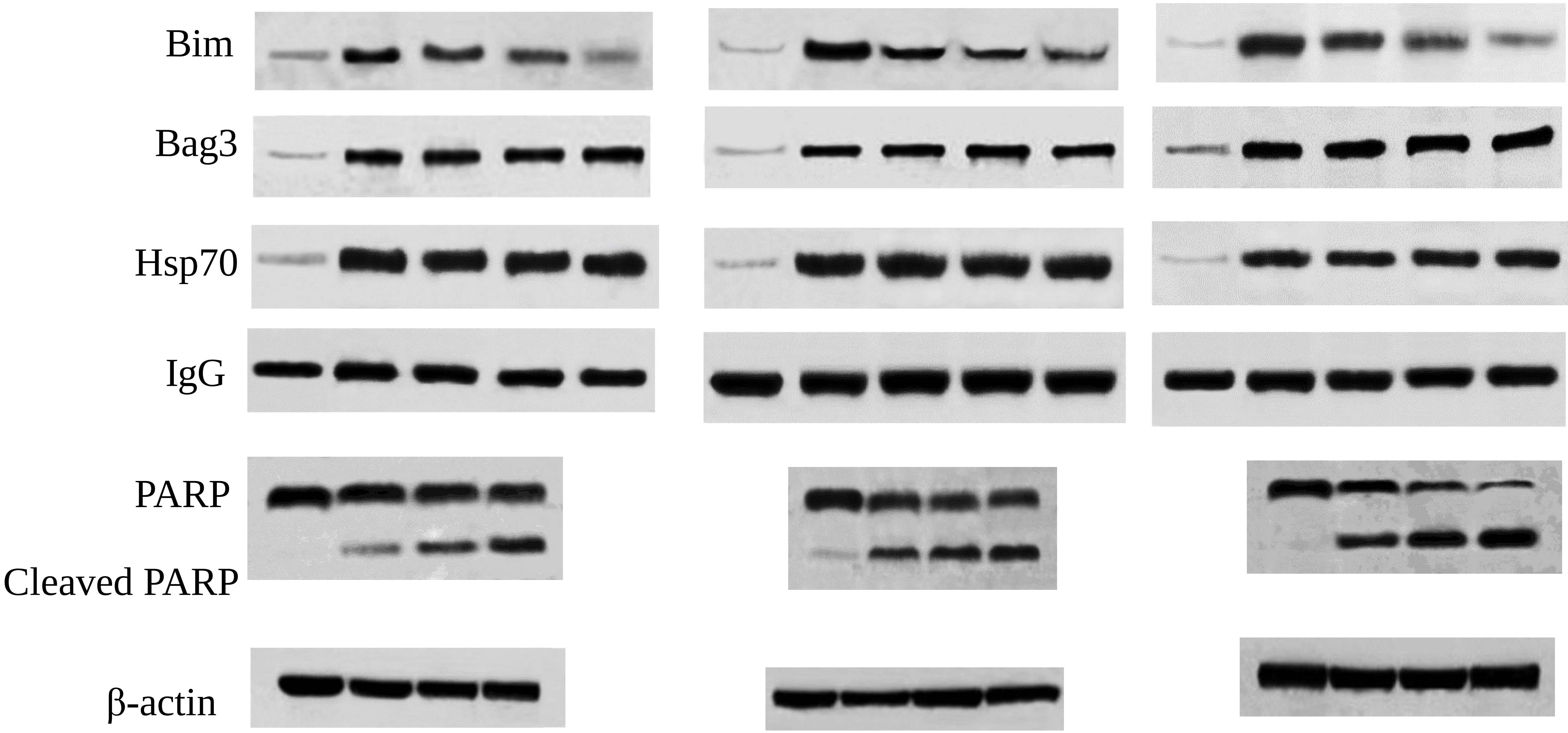

Fig. 3F

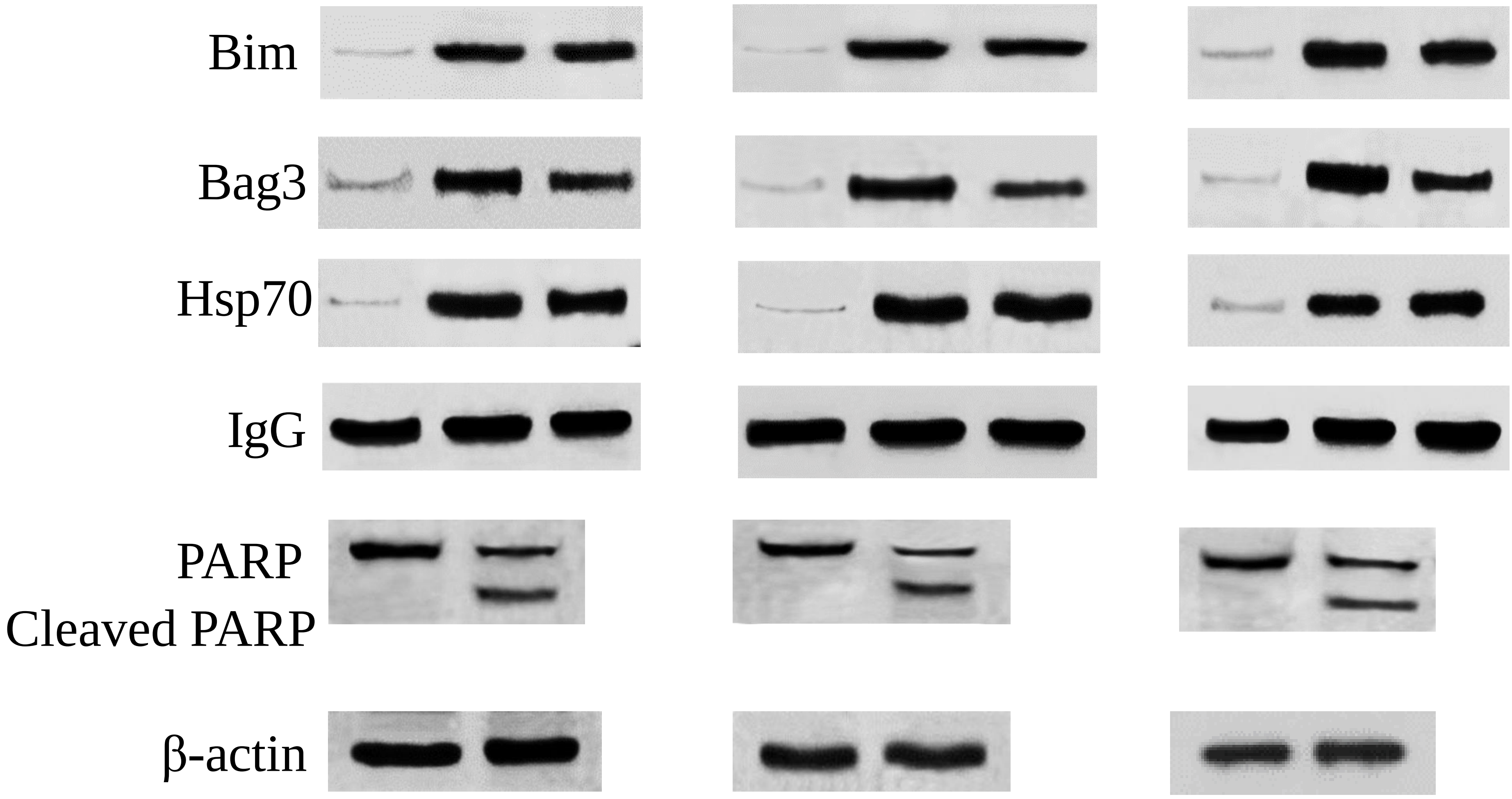

Fig. 4A

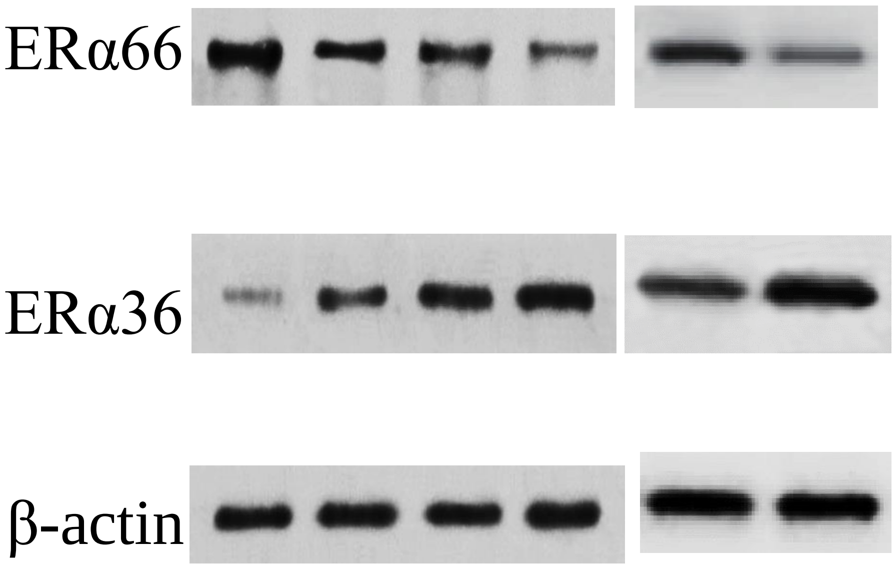

Fig. 4B

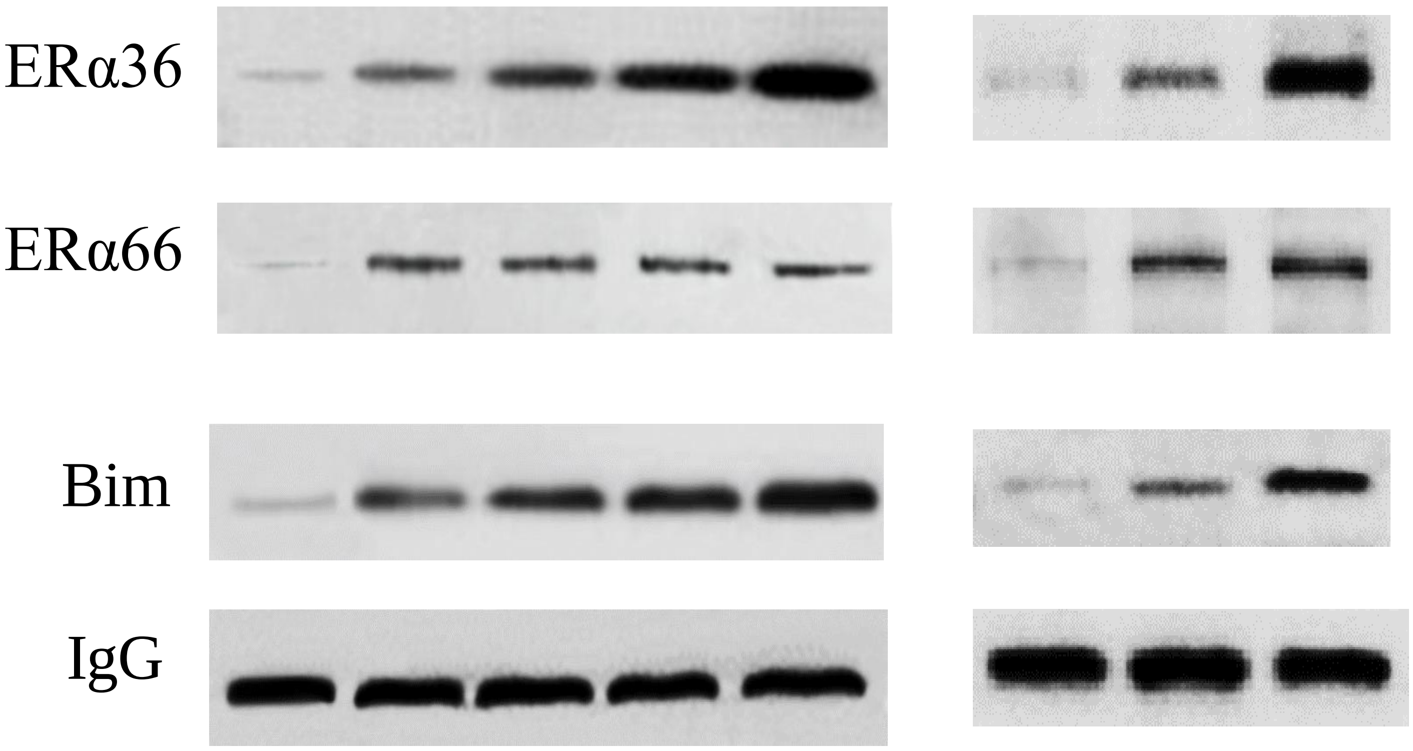

Fig. 4C

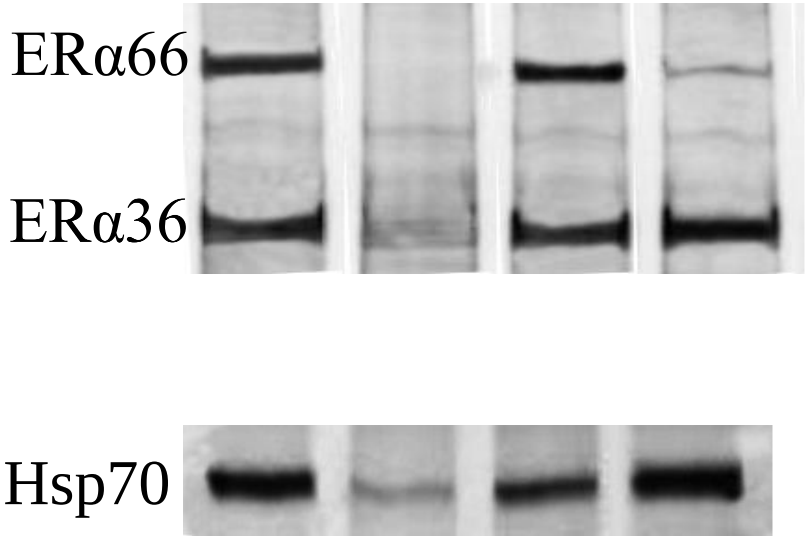

Fig. 4D

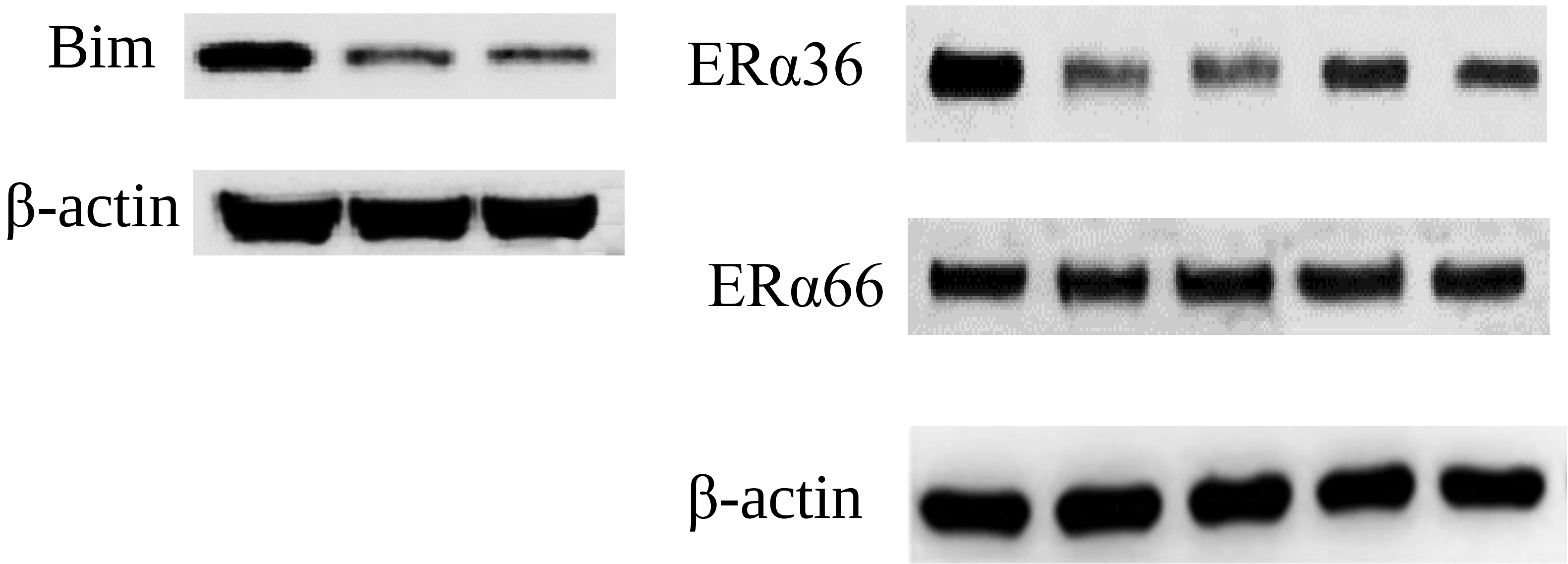

Fig. 4E

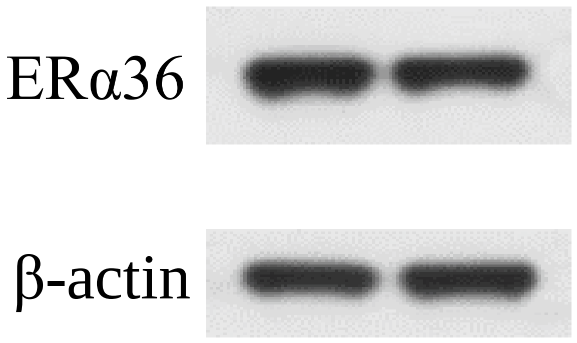

Fig. 5A

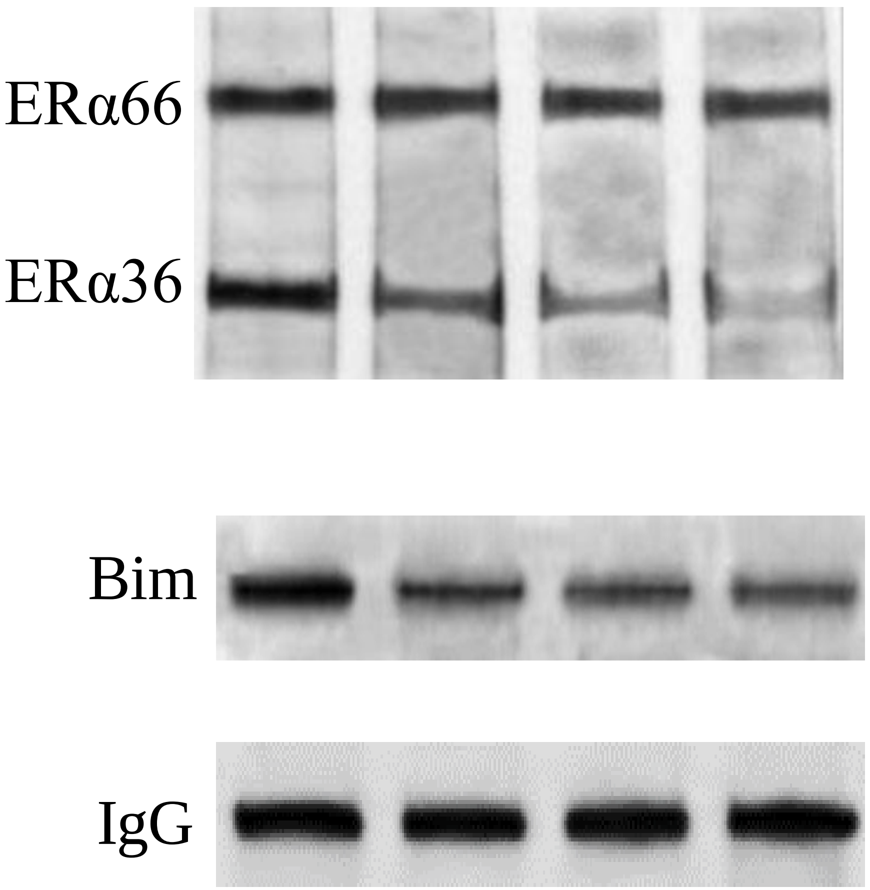

Fig. 5B

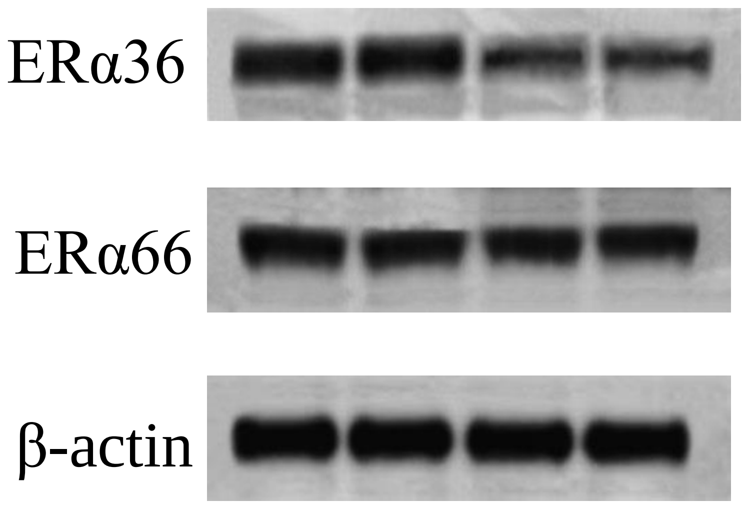

Fig. 5C

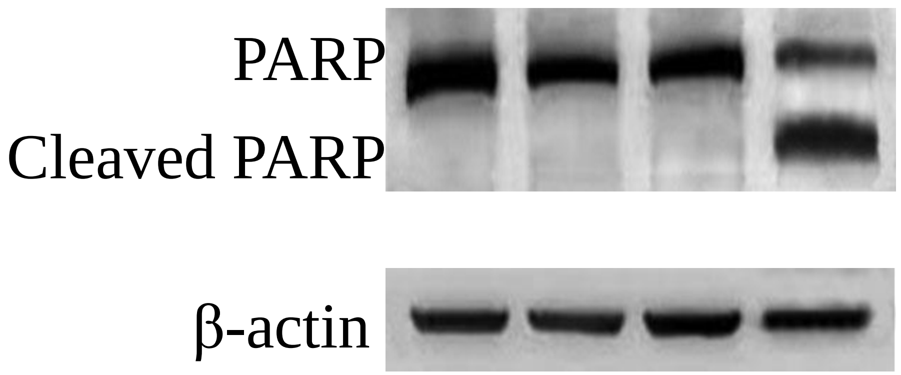

Fig. 5D

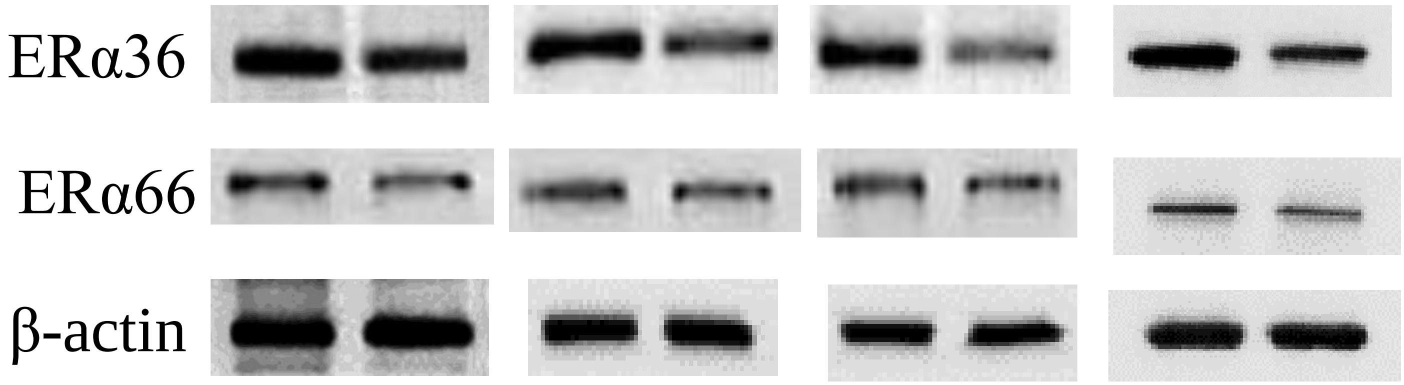

Fig. 5E

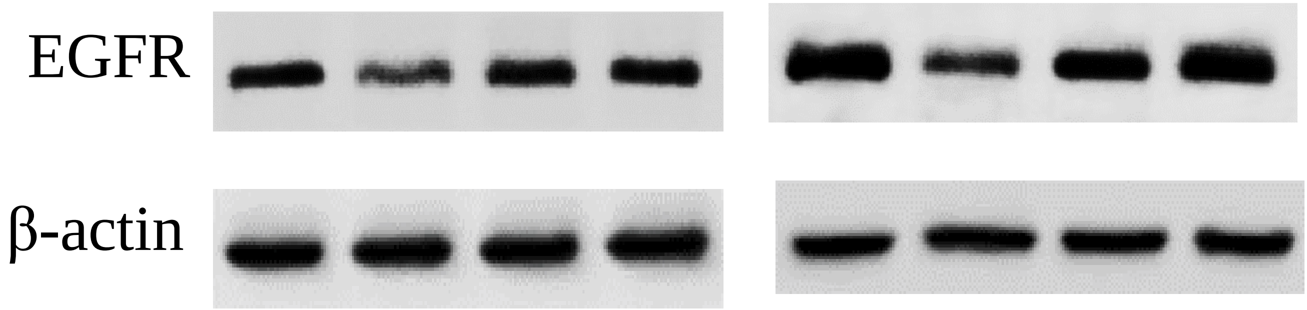

Fig. 6E

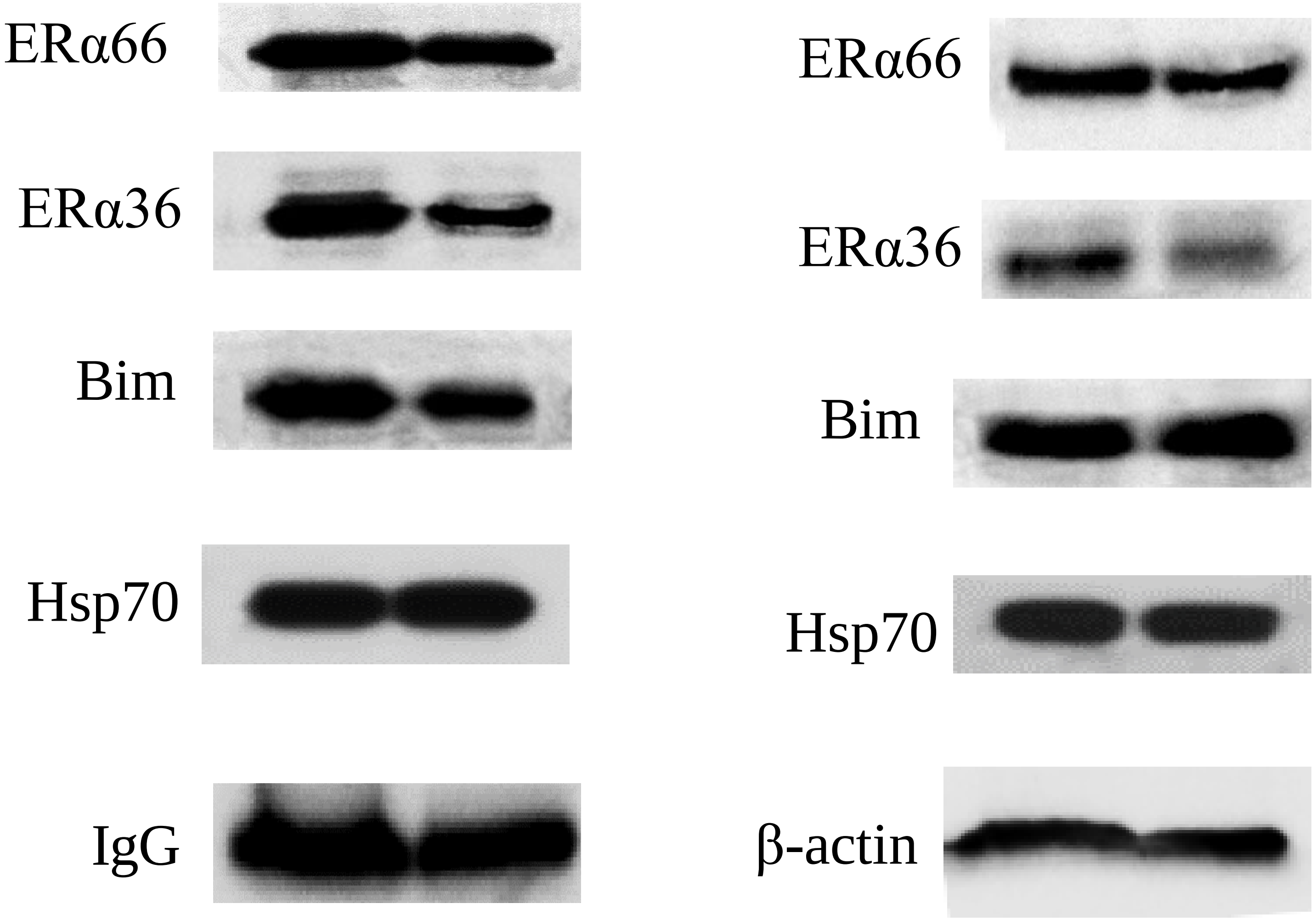

Supplement: Supplementary file 1 — Additional file 1. Original western blot. [file 13058_2024_1790_MOESM1_ESM.pdf]
