# Supplementary material for: Small molecule inhibitor targeting the Hsp70-Bim protein–protein interaction in estrogen receptor-positive breast cancer overcomes tamoxifen resistance
Source: Breast Cancer Res. 2024 Feb 26;26:33. doi: 10.1186/s13058-024-01790-0 (PMC10895875; doi:10.1186/s13058-024-01790-0)
Supplement: Supplementary file 2 — Additional file 2. Supplementary Figures and Tables. [file 13058_2024_1790_MOESM2_ESM.docx]

**Material and methods**

**Apoptosis assay**

Apoptosis was quantified by surface Annexin V-FITC staining. Cells were treated with inhibitor in 48 hr and then transferred from a culture well (1.2 × 10^6^/well) to a tube and

washed with PBS containing 1% (v/v) bovine calf serum (Hyclone, Logan, UT, USA). According to the manufacturer’s instructions, the cells were incubated with a 1:40 solution of FITC-conjugated Annexin V (Roche Diagnostics, Germany) in the dark for 10 min at room temperature and the Annexin V-FITC positive cells were analyzed by flow cytometry on a BD FACSCalibur (BD Biosciences, Becton Drive, Franklin Lakes, NJ, USA). Cell Questc software (BD Biosciences) was used to determine the percentage of apoptosis in the samples.

**Fig. S1.** MCF-7, MCF-7/TAM-R, T47D and T47D/TAM-R were treated with a gradient concentration of **MKT-077**, **VER-155008** and **S1g-2** alone or in combination with **tamoxifen** at constant ratios spanning the IC_50_ dose of each agent for 48 hr followed by CCK8 assay. The data show mean ± SD (n=3 biologically independent experiments).

**Fig. S2.** Western blot analysis of the levels of Hsp70 in MCF-7, MCF-7/TAM-R, T47D and T47D/TAM-R cells. The graphs show (mean ± SD, n=3 biologically independent experiments) protein level in MCF-7/TAM-R, T47D and T47D/TAM-R cells normalized to that in MCF-7.

**Fig. S3. Bio-informatic analysis of Hsp70 expression and its association with survival.** (A) Hsp70 mRNA expression between healthy tissue (breast) and cancer tissue (invasive breast cancer). Data was obtained from GEPIA2 database. (B) DRUGSURV Database shows that high levels of HSPA1A mRNA predicts a poor survival compared to low levels in patients suffering from breast cancer.


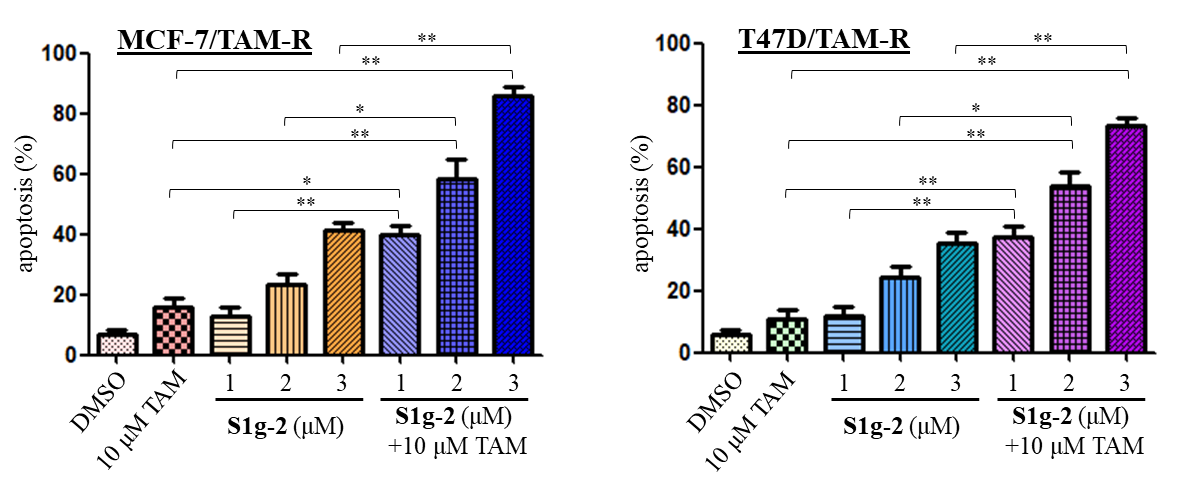


**Fig. S4. S1g-2 enhanced tamoxifen-induced apoptosis in resistant cell lines.** MCF-7/TAM-R or T47D/TAM-R were treated with 10 μM **tamoxifen**, 1, 2 or 3 μM **S1g-2** alone or in combination for 48 hr, followed by apoptosis assay by Annexin V staining. ^*^*P*<0.05, ^**^*P*<0.01 (one-way ANOVA test).

**Fig. S5. S1g-2 induced disruption of Hsp70-Bim PPI at 12 hr and apoptosis at 24 hr.** (A) co-IP analysis of the levels of Hsp70-Bim PPI in MCF-7/TAM-R cells upon treatment with10 μM **S1g-2** treatment for 0, 6, 12 or 24 hr. (B) western blot analysis of the levels of PARP cleavage in MCF-7/TAM-R upon 10 μM **S1g-2** treatment for 0, 6, 12 or 24 hr, using β-actin as a loading control.

**Table S1**. The IC_50_ value of **MKT-077**, **S1g-2**, **VER-155008** and **tamoxifen** for cell killing activity in the **tamoxifen**-sensitive and -resistant breast cancer cell lines


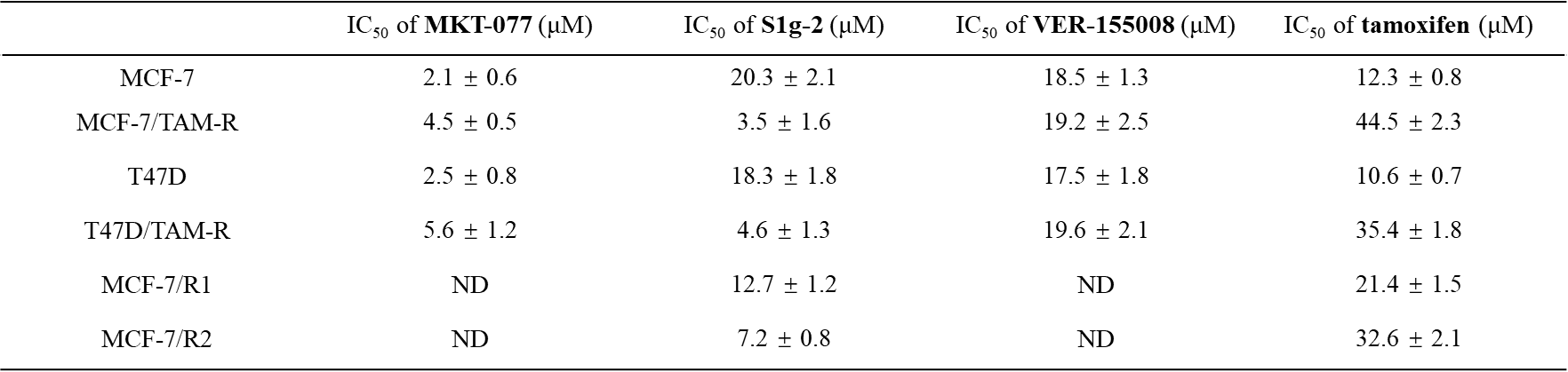


ND indicates not determined.

**Table S2**. The potency of **MKT-077**, **S1g-2** and **VER-155008** respectively in combinations with **tamoxifen**

Note: To measure CI index values, cells were simultaneously treated with **MKT-077** and **tamoxifen** at 1:5 for MCF-7, 1:10 for MCF-7/TAM-R, 1:5 for T47D, 1:6 for T47D/TAM-R; **S1g-2** and **tamoxifen** at 2:1 for MCF-7, 1:10 for MCF-7/TAM-R, 2:1 for T47D, 1:8 for T47D/TAM-R; **VER-155008** and **tamoxifen** at 2:1 for MCF-7, 1:2 for MCF-7/TAM-R, 2:1 for T47D, 1:2 for T47D/TAM-R. CI < 1, CI = 1 and CI > 1 indicate synergism, addictive effect, and antagonism, respectively (column 2, 4 and 6). When 50% growth inhibition level was induced by two drugs combination at constant ratios, the respective doses of the two drugs are presented as mean ± SD, n = 3 (column 1, 3 and 5).
